# Thermodynamic criteria of the end-of-life silicon wafers refining for closing the recycling loop of photovoltaic panels

**DOI:** 10.1080/14686996.2019.1641429

**Published:** 2019-07-10

**Authors:** Xin Lu, Takahiro Miki, Osamu Takeda, Hongmin Zhu, Tetsuya Nagasaka

**Affiliations:** Graduate School of Engineering, Tohoku University, Miyagi, Japan

**Keywords:** Thermodynamic criteria, silicon refining, end-of-life wafer, photovoltaic panels, pyro-metallurgical recycling, solvent refining, high-temperature purification, 50 Energy Materials, 308 Materials resources / recycling

## Abstract

The collected end-of-life (EoL) silicon wafers from the discharged photovoltaic (PV) panels are easily contaminated by impurities such as doping elements and attached materials. In this study, the thermodynamic criteria for EoL silicon wafers refining using three most typical metallurgical refining processes: oxidation refining, evaporation refining, and solvent refining were systemically and quantitatively evaluated. A total of 42 elements (Ag, Al, Au, B, Be, Bi, C, Ca, Ce, Co, Cr, Cu, Fe, Ga, Gd, Ge, Hf, In, La, Mg, Mn, Mo, Na, Nb, Ni, Os, P, Pb, Pd, Pt, Re, Ru, Sb, Sn, Ta, Ti, U, V, W, Y, Zn, Zr) that are likely to be contained in the collected EoL silicon-based PV panels were considered. The principal findings are that the removal of aluminum, beryllium, boron, calcium, gadolinium, hafnium, uranium, yttrium, and zirconium into the slag, and removal of antimony, bismuth, carbon, lead, magnesium, phosphorus, silver, sodium, and zinc into vapor phase is possible. Further, solvent refining process using aluminum, copper, and zinc as the solvent metals, among the considered 14 potential ones, was found to be efficient for the EoL silicon wafers refining. Particularly, purification of the phosphorus doped *n*-type PV panels using solvent metal zinc and purification of the boron doped *p*-type PV panels using solvent metal aluminum are preferable. The efficiency of metallurgical processes for separating most of the impurity elements was demonstrated, and to promote the recycling efficiency, a comprehensive management and recycling system considering the metallurgical criteria of EoL silicon wafers refining is critical.

## Graphical Abstract

**Figure:**
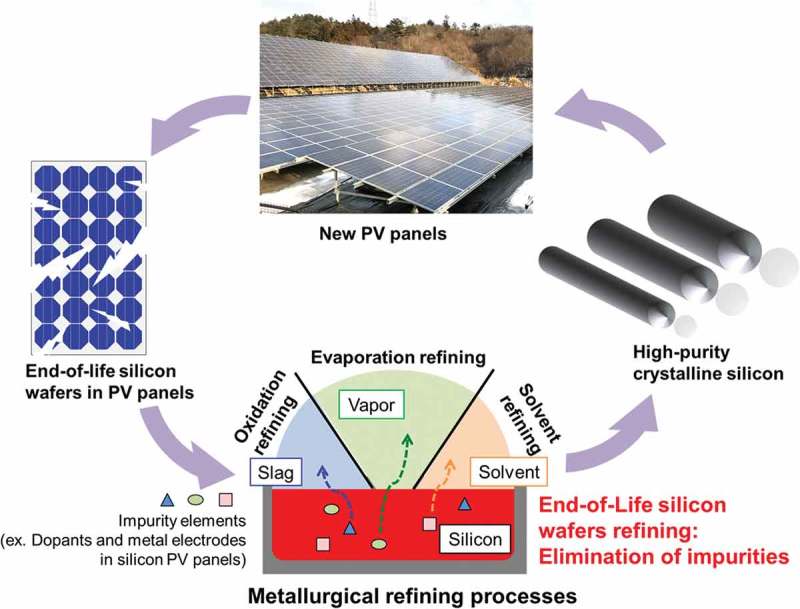

## 1 Introduction

**Figure 1.**
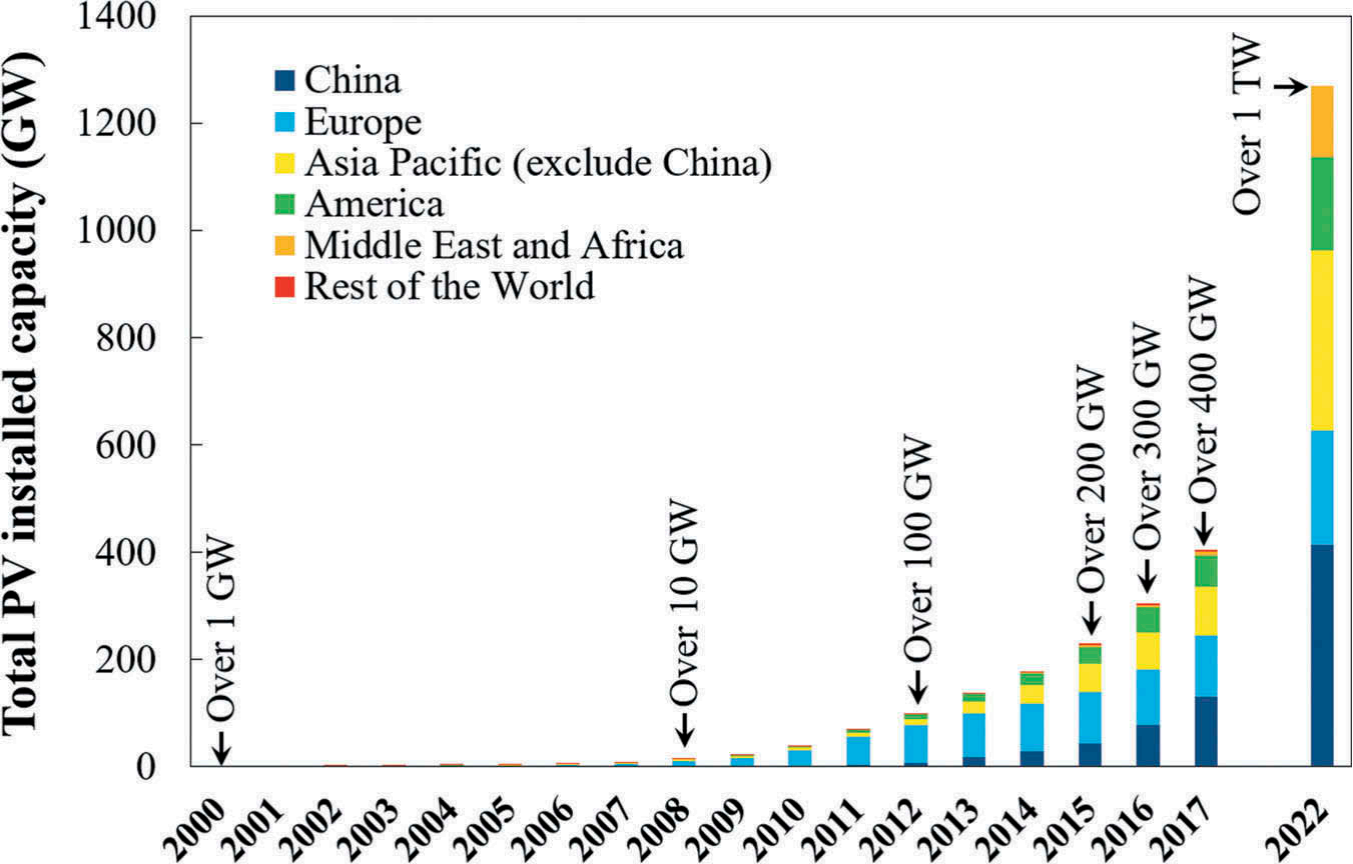
The global total capacity of solar PV installed from 2000 to 2017 and the forecast capacity by 2022 under optimal conditions [3].

As a clean and renewable energy source, solar energy shows great environmental advantages compared with fossil-fuel energy, such as reducing greenhouse gas (GHG) emissions, air and water pollution, and saving natural energy resources [1,2]. The usage of solar energy based on photovoltaic (PV) technology has skyrocketed during the past decade, as shown in Figure 1 [3]. The total capacity of solar PV installed reached 404.5 GW by the end of 2017, which increased over 300 times from the beginning of the century (2000: 1.3 GW) [3]. Moreover, this value is expected to exceed 500 GW in 2018, and reach terawatt (TW) level (1270.5 GW) by the end of 2022 [3]. The PV panels installed generally have a long service life of 25–30 years, and the first batch of PV panels installed are now being retired. Given the growth of the PV market, the cumulative amount of end-of-life (EoL) PV panels was estimated to be over 70 million tonnes in 2050 [4].

The coming boom of the EoL PV panels presents another environmental issue because they are classified as hazardous and toxic waste [2,5]. The EoL PV panels contain heavy metals, such as lead, tin, and cadmium, which cause environmental pollution and pose threats to human health [5]. The European Union recently revised the European Waste Electrical and Electronic Equipment (WEEE) Directive, where EoL PV panels were added under the category of discharged electrical/electronic waste [6]. Henceforth, it is mandatory to define alternative strategies to landfilling, and recycling the EoL PV panels has become an obligation. On the other hand, recycling of EoL PV panels shows great environmental advantages, such as energy conservation, CO_2_ emissions reduction, natural resources conservation, reduction of landfilling, and reduction of heavy metals pollution [2,4,5,7].

**Figure 2.**
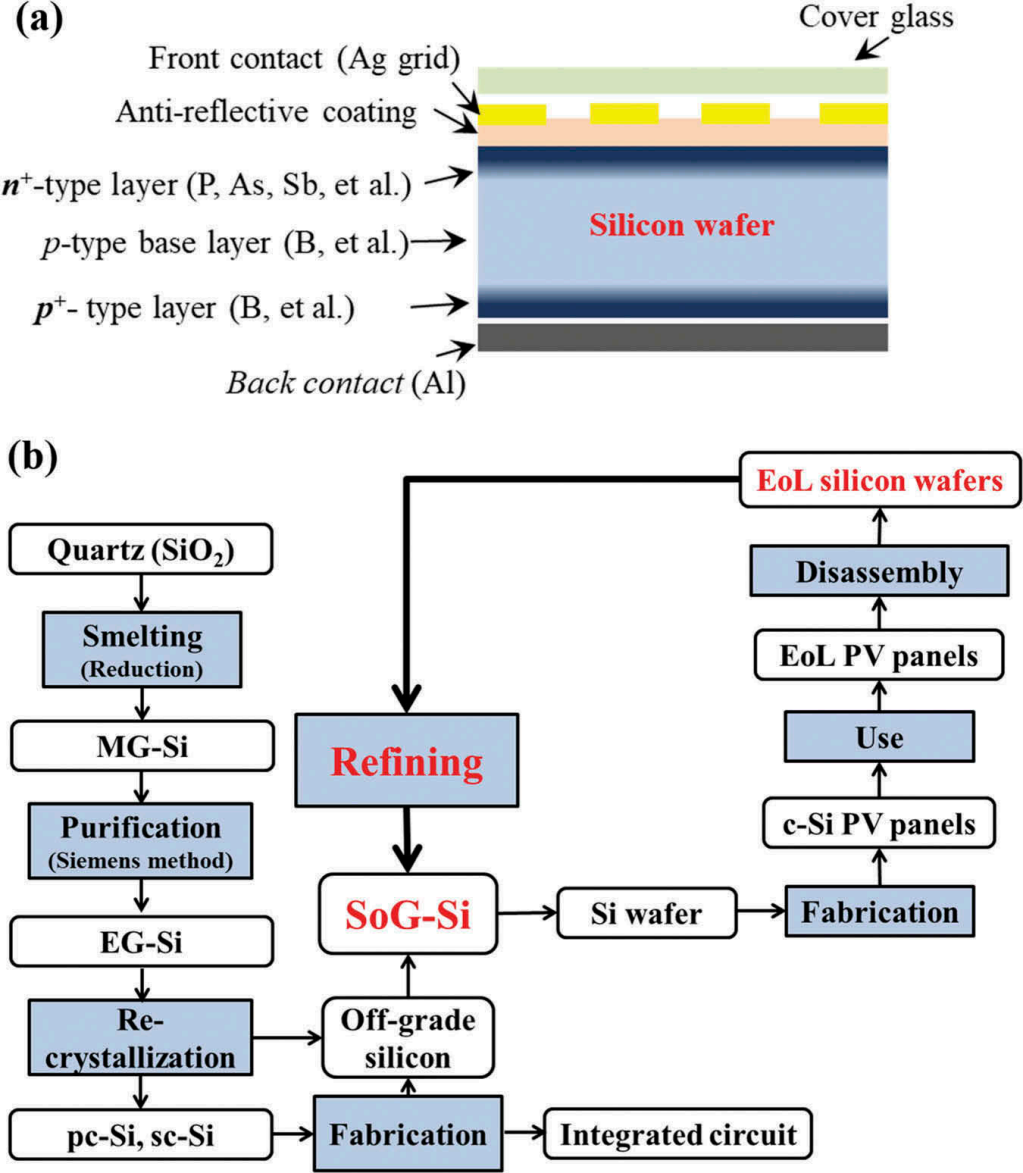
(a) A typical structure of c-Si PV cell, and (b) a schematic recycling loop of the silicon wafers from EoL PV panels. MG-Si: Metallurgical grade silicon; EG-Si: electronic grade silicon; pc-Si: poly-crystalline silicon; sc-Si: single-crystalline silicon; SoG-Si: Solar grade silicon; EoL: end-of-life; PV panels: photovoltaic panels.

Developing an efficient recycling system is becoming crucial to face the coming boom of the EoL PV panels [4,5,8]. By now, over 90% of the global PV market is dominated by the crystalline silicon (c-Si)-based PV panels, where the silicon wafer is the most important core part [9]. The silicon wafer, as shown in Figure 2(a), produced from solar-grade silicon (SoG-Si) (>99.9999% purity), is typically a moderately doped *p*-type c-Si semiconductor with a heavy doped *n* ^+^-type layer on the top side and heavy doped *p* ^+^-type layer on the back side. Figure 2(b) schematically shows the recycling loop of silicon wafers from EoL c-Si PV panels for new SoG-Si. The recycling process of the EoL c-Si PV panels starts from the disassembly of the sandwich layer-like structure of the EoL silicon wafers. The silicon wafers can be separated by methods such as mechanical crushing, pyrolysis, organic solvent, or acid etching to remove the encapsulant and electrode materials [4,5,8,10]. The wafers obtained without any damages can be reused directly, while most of them with edge chippings, micro-cracks, or electronic damages would be subject to a refining process to recycle the silicon metal [11,12].

**Figure 3.**
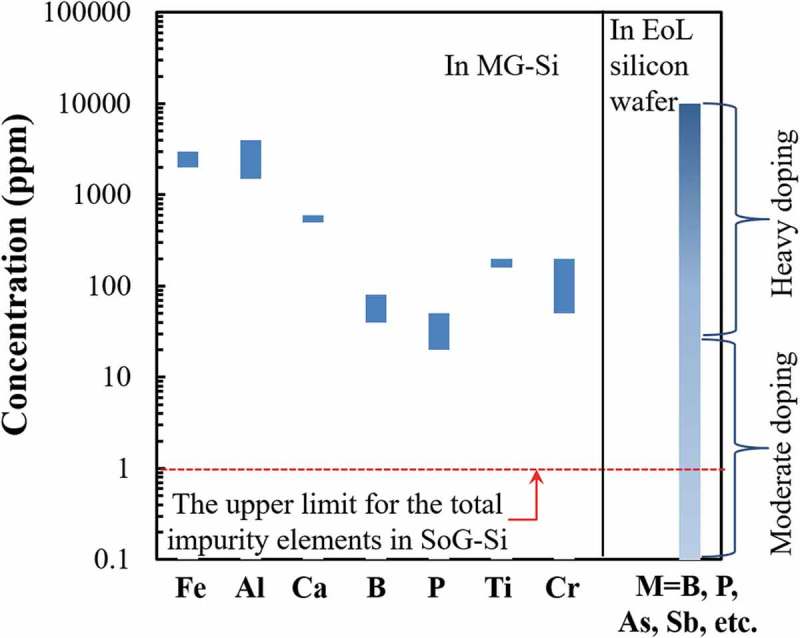
Concentrations of typical impurity elements in MG-Si and in the EoL silicon wafer from PV panels. M: the impurity elements in EoL silicon wafer.

Refining the EoL silicon wafers becomes the key to close the recycling loop of the PV panels [13–15]. Figure 3 compares the concentrations of typical impurity elements in EoL silicon wafers and metallurgical-grade silicon (MG-Si), the raw materials with purity of approximately 98% produced by reducing quartz from natural ore [16,17]. The typical impurity elements in MG-Si are aluminum, iron, titanium, and calcium with relatively fixed concentration. On the other hand, the impurity elements in the EoL silicon wafer are mainly from the doping elements used to form the semiconductors. The typical doping elements include the group IIIA atoms as acceptors for *p*-type semiconductors, such as boron, and group VA atoms as donors for *n*-type semiconductors, such as phosphorus, arsenic, and antimony [17,18]. The mixture of different types of wafers and the incomplete separation of the attached materials, such as aluminum alloy frame, silver grid line, and tin or copper wire, make the composition of the collected EoL c-Si products more complicated and variable than that of MG-Si. The contamination by these impurity elements dramatically downgrades the recycled silicon. Thus, the impurity elements must be eliminated before the use of silicon in new PV panels [13–15,19].

Chemical etching using solutions such as HNO_3_+ HF + CH_3_COOH + Br_2_ or HF + HNO_3_ + H_2_SO_4_ + CH_3_COOH + CMP-MO-2 (surfactant) has been attempted to remove the metal impurities such as metal electrodes and *n-p* junction [10,20,21]. However, long treatment duration and a complicated hazardous acid mixture discharge make it difficult to be employed in practical industrial applications. On the other hand, the metallurgical refining process, which has been attempted for the MG-Si purification (Supporting Information) [16,22–25], is simple and well-established. The metallurgical refining process is also a promising method for purification of the EoL silicon wafers as the new resource for SoG-Si in new PV panels.

This study is meant to systemically examine the thermodynamic criteria of the metallurgical refining process of the EoL silicon wafers for closing the recycling loop of EoL c-Si PV panels. The elimination limitation of impurity elements by three most typical metallurgical refining processes: oxidation refining, evaporation refining, and solvent refining, was quantitatively evaluated using a developed thermodynamic method [26–33]. A total of 42 impurity elements that are likely to be present in the collected EoL PV panels were considered. All the thermodynamic parameters were used in the evaluation, and the influence of the physico-chemical factors including temperature, oxygen partial pressure, and slag composition was extensively examined.

## 2 Materials and methods

In previous studies, a thermodynamic method to evaluate the elimination limit of impurity elements contained in the EoL products of iron/steel, copper, zinc, lead, aluminum, magnesium, titanium, and Ni-Co-based superalloys was proposed [26–33]. This thermodynamic method was developed and used to evaluate the criteria of the EoL silicon wafers refining during the EoL PV panels recycling.

### 2.1 Oxidation refining and evaporation refining

The re-melting process of EoL silicon wafers with oxide-slag treatment was simulated. The elimination of a given impurity element, M, can be quantitatively presented by its equilibrium distribution ratio between molten silicon and the oxide slag (*L* ^slag/silicon^), and that between molten silicon and the gas phase (*L* ^gas/silicon^) [26–33]. *L* ^slag/silicon^ was defined as the ratio of the molar fractions of elements in slag over that in silicon, while *L* ^gas/silicon^ was defined as the ratio of the partial pressure of elements in gas phase and that of silicon solvent. The calculation methods were shown as followings.

(1) $$M\left(l\right)=M\left(g\right)$$

(2) $$L^{gas/silicon}=\frac{p_{M}/p_{\;}^{o}}{p_{Si}/p_{\;}^{o}}=\frac{p_{M}^{o}\gamma_{M}(l)x_{M}(l)}{p_{Si}}$$

(3) $$M(l)+\frac{n}{2}O_{2}(g)=MO_{n}(slag)$$

(4) $$L^{slag/silicon}=\frac{x_{MO_{n}}}{x_{M}(l)}=\frac{K_{3}\gamma_{M}^{\;}(l){\left(p_{O_{2}}/p_{\;}^{o}\right)}^{n/2}}{\gamma_{MO_{n}}}$$

where $p_{M}^{o}$, $p_{M}$, and $p_{Si}$ are the partial pressures of pure M (Pa), of M dissolved in the molten silicon (Pa), and of the molten silicon (Pa); $a_{M}^{\;}(l)$,$\gamma_{M}^{\;}(l)$, and $x_{M}^{\;}(l)$ are the activity and activity coefficient of M refer to the pure liquid standard state, and the mole fraction of M in molten silicon, respectively; $\gamma_{MO_{n}}^{\;}$and $x_{MO_{n}}^{\;}$ are the activity coefficient of the oxide product MO_n_ in the pure standard state and its mole fraction in the slag phase; $p_{O_{2}}^{\;}$and $p_{\;}^{o}$ are the oxygen partial pressure (Pa) and the conversion factor (101,325 Pa/atm). The symbols l, g, and slag denote the state of the species in the liquid silicon, in the gas phase, and in the slag, respectively. *K*_3_ is the equilibrium constant of Equation (3) and is calculated based on the change in the Gibbs free energy of Equation (3).

#### 2.1.1 Temperature, free energy and vapor pressure

The temperature of the simulated remelting process was set to 1773 K as a base case. Thermodynamic data are available for the changes in the standard Gibbs free energy for evaporation and oxidation of the pure element M (Equations (1) and (3)) [34]. The molar fraction of M was set at 0.0001 as the first approximation for the calculation of vapor pressure.

#### 2.1.2 Activity coefficients of impurity elements in molten silicon

The activity coefficients of impurity elements in molten silicon were calculated based on the Redlich-Kister polynomial, which is a typical method used in the CALPHAD (CALculation of PHAse Diagrams) (Supporting Information) [35]. The activity coefficient of impurity element M in the binary Si-M system can be calculated by the following polynomials:

(5) $$\begin{array}{rl} & RT\ln\gamma_{M}{=}^{0}\Omega_{Si-M}x_{Si}^{2}{+}^{1}\Omega_{Si-M}x_{Si}^{2}(4x_{Si}-3)\\ & \;\;\;\;\;\;\;\;\;\;\;\;\;\;\;\;\;\;\;\;\;\;\;\;{+}^{2}\Omega_{Si-M}x_{Si}^{2}(2x_{Si}-1)(6x_{Si}-5)\\ & \;\;\;\;\;\;\;\;\;\;\;\;\;\;\;\;\;\;\;\;\;\;\;\;{+}^{3}\Omega_{Si-M}x_{Si}^{2}(2x_{Si}-1{)}^{2}(8x_{Si}-7) \end{array}$$

Since the concentration of impurity elements in silicon is extremely low in most cases, it is more accurate to use the activity coefficients of M in the dilute solution, $\gamma_{M}^{0}$. $\gamma_{M}^{0}$ can be calculated much more simply at the limiting case of $x_{Si}≅1$:

(6) $$RT\ln\gamma_{M}^{0}=\sum_{p=0}^{3}{\;}^{p}\Omega_{Si-M}$$

where ${\;}^{p}\Omega_{Si-M}$(*p *= 0, 1, 2, 3) are temperature-dependent interaction parameters.

The selection of the assessed interaction parameters for the liquid Si-M systems mainly refers to the review on the thermodynamics of elements in the dilute silicon melts by the authors [36].

#### 2.1.3 Activity coefficients of the oxides of impurity elements in slag

In practical metallurgical remelting process, the slag is always designed to control the activity coefficients of the oxide product in the slag. For this reason, the activity coefficients of oxides in slag have been extensively studied. In this study, the activity coefficient of MO_n_ in slag was assumed to be unity as a first approximation [26–33]. Further, the effects of the activity coefficient of the oxide product on the elimination of impurity elements will be discussed.

#### 2.1.4 Oxygen partial pressure

The oxygen partial pressure of the simulated remelting process was determined by the equilibrium oxygen partial pressure between pure molten silicon ($a_{Si}=1$) and the pure solid SiO_2_ ($a_{SiO_{2}}=1$) in slag, which was calculated as 3.61 × 10^−13^ ****Pa for the simulated remelting process at 1773 K based on the thermodynamic data [34]. The oxygen partial pressure presented here is a critical value, and significant oxidation loss of silicon occurs once above this value.

### 2.2 Solvent refining

Solvent refining eliminates impurity elements according to their different affinities with solid silicon and molten solvent metals. The elimination of a given impurity element, M, by the solvent refining process is controlled by its equilibrium distribution ratio between solid silicon and molten solvent metals (*L* ^solvent/silicon^). *L* ^solvent/silicon^ was defined as the ratio of the molar fractions of elements in the solvent melts over that in solid silicon, and calculated as followings.

(7) $$M({s)}^{in\;solid\;silicon}=M({l)}^{in\;molten\;solvent}$$

(8) $$K_{5}=\frac{a_{M}(sol.)}{a_{M}(s)}=\frac{x_{M}(sol.)\gamma_{M}(sol.)}{x_{M}(s)\gamma_{M}(s)}$$

(9) $$L_{\;}^{solvent/silicon}=\frac{x_{M}(sol.)}{x_{M}(s)}=\frac{K_{5}\gamma_{M}(s)}{\gamma_{M}(sol.)}$$

where, $a_{M}(sol.)$, $\gamma_{M}(sol.)$, and $x_{M}(sol.)$ are the activity and activity coefficient refer to the pure liquid standard state and the mole fraction of M in the liquid solvent metal; $a_{M}(s)$, $\gamma_{M}(s)$, and $x_{M}(s)$ are the activity and activity coefficient in the pure solid standard state and the mole fraction of M in solid silicon phase.

#### 2.2.1 Temperature and free energy

The temperature of the simulated solvent refining process was set according to the eutectic temperature of the binary system of silicon the used solvent metal. Thermodynamic data are available for the changes in the standard Gibbs free energy for conversion of the state of M (Equation (5)) [37].

#### 2.2.2 Activity coefficients of impurity elements in solid silicon and in solvent metals

The same calculation method based on Redlich-Kister polynomial as that shown in Section 2.1 was used to calculate the activity coefficients of impurity elements in solid silicon and candidate solvent metal melts (aluminum, copper, iron, lead, tin, and zinc) (Supporting Information).

The selection of the assessed interaction parameters for the solid Si-M systems mainly refers to the review work by Yoshikawa et al [38].

## 3 Results and discussion

### 3.1 Purification of silicon wafers from EoL PV panels by oxidation refining and evaporation refining

**Figure 4.**
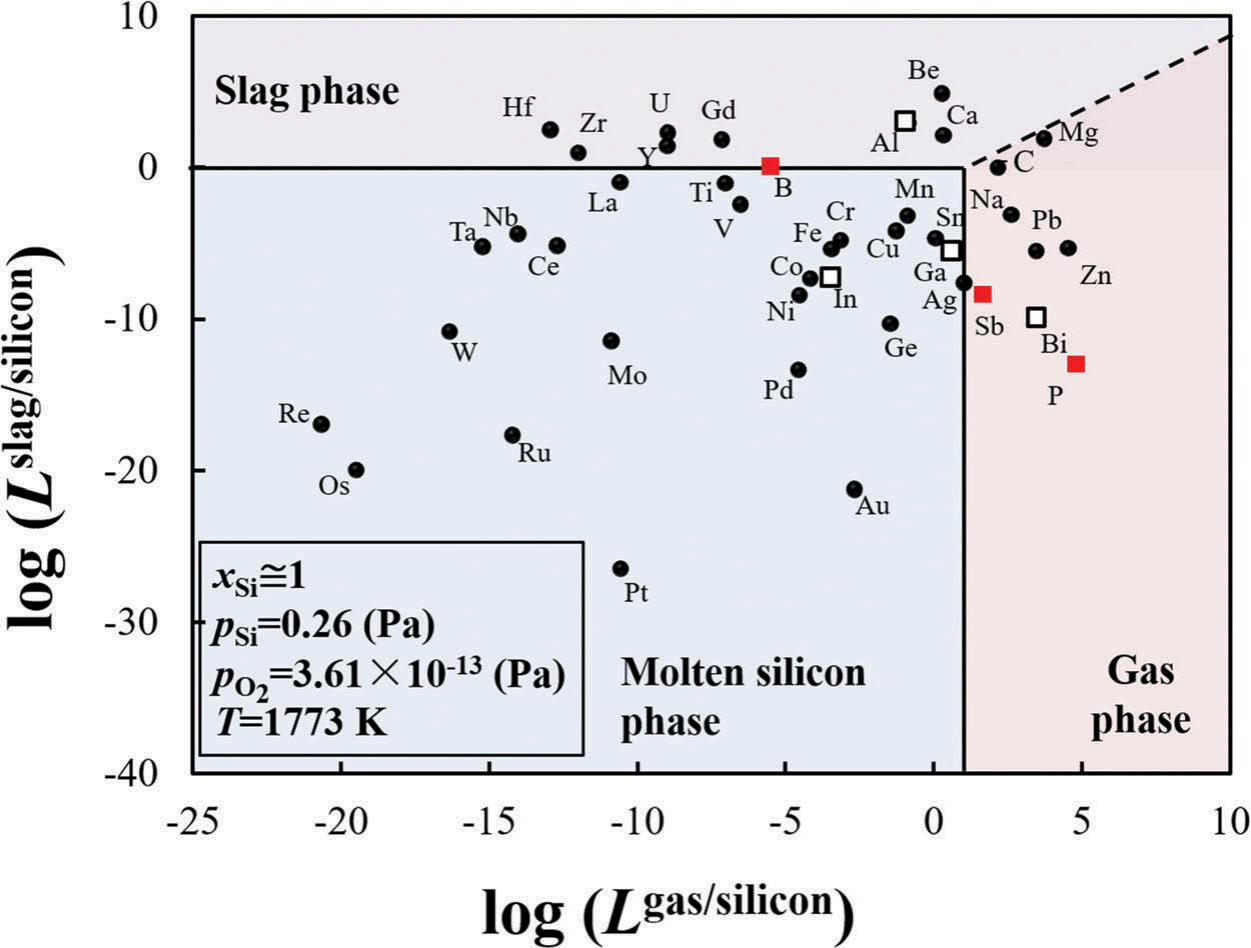
Elimination limit of impurity elements during recycling of EoL silicon wafers using the re-melting process. (Red squares: the most used dopants; open squares: other elements from the Groups IIIA and VA.).

The distribution tendencies of the 42 impurity elements likely to be present in the EoL PV panels, among the molten silicon, slag, and gas phases during re-melting process are shown in Figure 4. The most used dopant elements in the silicon wafers, boron, phosphorus, and antimony, are highlighted as red squares, while other elements from groups IIIA and VA are represented as open squares. The boundary between the molten silicon and gas phase is assumed to satisfy the condition log(*L* ^gas/silicon^) = 1. In this boundary condition, the number of M atoms is 10 times as much as the number of silicon atoms in the gas phase and thus appears to be adequate for preferential removal of M into the gas phase by evaporation. The boundary between the molten silicon and slat phase is assumed to satisfy the condition log(*L* ^slag/silicon^) = 0. It is considered that in the industrial sense the removal of M into slag phase can be promoted by increasing the slag amount, repeating the oxidation refining process, and selecting the suitable slag composition.

During the recycling of EoL silicon wafers by re-melting process, aluminum, beryllium, calcium, gadolinium, hafnium, uranium, yttrium, and zirconium can be removed from molten silicon into the slag phase by oxidation. The removal of boron which has an almost equal distribution tendency into slag and the metal phases by slag treatment might be possible if the slag is suitable. That is, the slag used must be capable of sufficiently reducing the activity coefficient of the formed boron oxide. Antimony, bismuth, carbon, lead, magnesium, phosphorus, silver, sodium, and zinc can be separated into the vapor phase by evaporation. Noted that removal of phosphorus in form of P_4_ (g), and removal of carbon in form of CO was considered (Supporting Information). On the other hand, the other considered 24 impurity elements have a strong tendency to remain in the molten silicon phase. These trends indicate that the removal of these impurity elements by either oxidation or by evaporation during the re-melting process is difficult. The results are in good agreement with the industrial measurement results reported by a practical MG-Si refining plant in Norway, which shows that the impurity elements of aluminum, calcium, beryllium, and magnesium, can be removed into the slag phase, while zinc, lead, and some magnesium can be removed into the gas phase [39].

The most important feature of Figure 4 is that it illustrates the metallurgical criteria of the refining of the EoL silicon wafer through remelting process. Attention must be paid to the impurity elements that remain in the metal phase during the recycling process. It is noted that the typical dopant elements in silicon semiconductors including phosphorus, boron, antimony, as well as aluminum, and bismuth, can be removed into slag phase or vapor phase by remelting process. However, removal of indium and gallium is difficult. Besides the dopant elements, silver, copper, and tin used as the metal electrodes on the front surface of the c-Si PV panels, might also be present in the collected EoL c-Si products [10]. Figure 4 shows that their removal is also difficult by re-melting process. Thus, complete separation of the metal electrodes is significantly important for closing the recycling loop of EoL c-Si PV panels by re-melting. The metallurgical criteria shown in the Figure 4 must be considered during the design of the EoL PV panels sorting system as well as during the creation of related policies to promote the resource efficiency of recycling.

#### 3.1.1 Effect of the temperature on the elimination limit of impurities by oxidation refining using slag treatment

**Figure 5.**
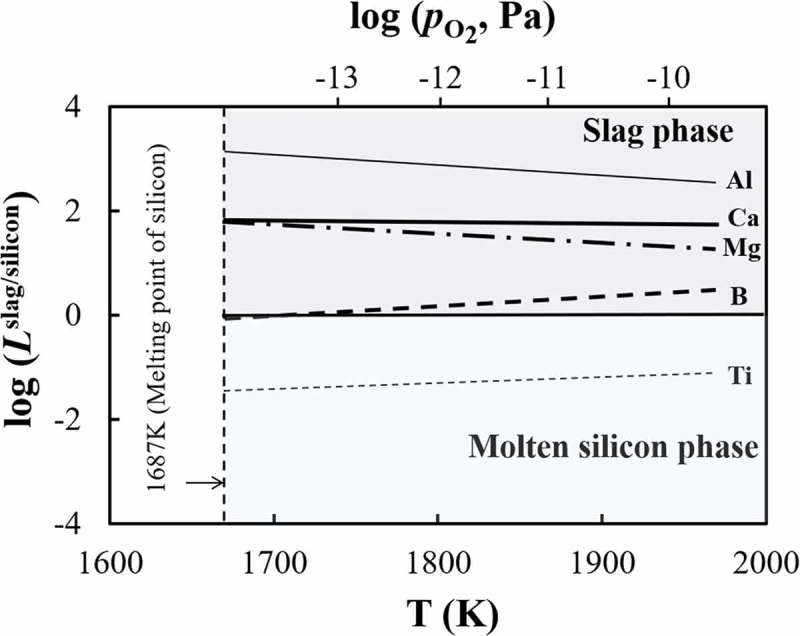
Temperature dependence of the distribution ratio between molten silicon phase and slag phase, *L* ^slag/silicon^.

As described in the Section 2, the elimination limit of an impurity element from silicon by oxidation is thermodynamically controlled by temperature, oxygen partial pressure, and the activity coefficient of the oxidation product in slag. Figure 5 shows the effect of temperature on the distribution tendencies of impurity elements between molten silicon and slag phases. Aluminum, boron, calcium, magnesium, and titanium, with distribution ratios *L* ^slag/silicon^ ranging from 10^4^ to 10^−4^ as shown in Figure 5, were selected. Two findings are worthy of note from Figure 5: (1) the effect of temperature on the distribution ratio between silicon melt and slag phase is not remarkably large; (2) the distribution ratio of boron increases and crosses the boundary of molten silicon phase and slag phase (*L* ^slag/silicon^ = 1 line) when temperature increases, which indicates that higher temperature is beneficial for boron removal using oxidation refining.

The equilibrium oxygen partial pressures between pure silicon and pure silicon oxide at different temperatures are also shown in the top of Figure 5. As mentioned above, a significant oxidation loss of the silicon to slag phase will occur above this critical value. In this sense, further improvements in the removal of impurity elements to the slag phase by increasing the oxygen partial pressure cannot be expected in practical refining process.

#### Effect of the slag composition ($\gamma_{MO_{n}}$) on the elimination limit of impurities by oxidation refining using slag treatment

3.1.2

Selection of a suitable slag to promote impurity elimination by reducing the activity coefficient of the oxide products of the impurity elements, $\gamma_{MO_{n}}$, is always an important task for metallurgists. For example, the $\gamma_{MO_{n}}$ for aluminum, calcium, magnesium, boron, and titanium in various slag, such as CaO-SiO_2_, CaO-SiO_2_-CaF_2_, CaO-Al_2_O_3_-SiO_2_, CaO-Al_2_O_3_-SiO_2_-Na_2_O, MgO-SiO_2_, and CaO-SiO_2_-MgO, and their distribution between slag and molten silicon has been experimentally investigated [40–46]. The effect of the slag composition on the $\gamma_{MO_{n}}$ can be largely attributed to the chemical properties of the oxide products. MgO and CaO are typical basic oxides, and always have a smaller $\gamma_{MO_{n}}$ than unity in the slag containing silicate owing to their strong affinity [40,45,46]. On the other hand, since BO_1.5_ and TiO_x_ are acidic oxides, their activity coefficients in slag with high concentration of CaO or MgO are also lower than unity [47–49].

**Figure 6.**
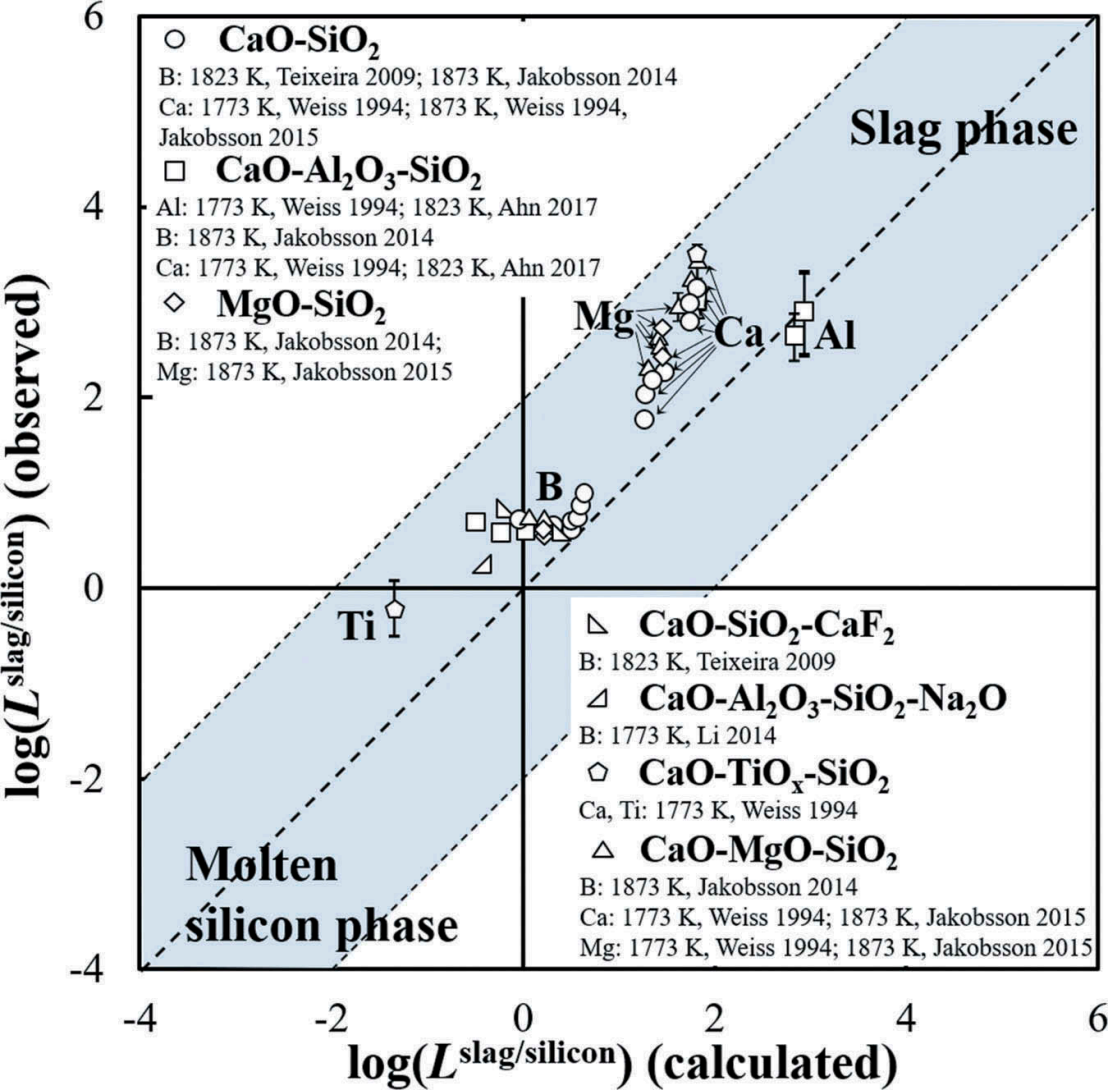
Comparison of the calculated distribution ratio, *L* ^slag/silicon^, with the experimentally observed results using different slag systems [40–46].

The effect of the slag composition on the removal of the impurity elements into slag phase is found to be insignificant, though the $\gamma_{MO_{n}}$ is variable according to the slag composition. Figure 6 compared the experimentally observed distribution ratios, *L* ^slag/silicon^, of aluminum, calcium, magnesium, boron, and titanium between the various practical slag and molten silicon with the calculated ones [40–46]. Some results from the literatures, in form of the ratio of the mass percentage of the elements in slag over that in silicon, were recalculated and shown in the form of the molar fraction ratio for the comparison purpose. The distribution tendency indicated by the *L* ^slag/silicon^ calculated by assuming $\gamma_{MO_{n}}$ as unity, as shown in Section 2, are found to agree well with the experimentally observed ones using practical slag (within ±2 order). Owing to the effect of the $\gamma_{MO_{n}}$ in the practical slag, the experimentally observed value of *L* ^slag/silicon^ for calcium, magnesium, boron, and titanium are all slightly higher than the calculated ones. Though the deviation exists, the distribution tendency for all the considered elements was not changed according to the variation of slag. Therefore, the effect of the slag composition on the removal tendency of the impurity elements is insufficient, and the evaluated results shown in Figure 4 can reasonably represent the distribution tendencies of impurity elements during the remelting of EoL silicon wafers. Notably, improvement of the slag composition can only work to promote the elimination of impurity elements distributed into slag phase, like aluminum, calcium, magnesium, and elements with *L* ^slag/silicon^ close to the distribution boundary, like boron (log(*L* ^slag/silicon^) = 0.1), but have no significant effect on the elimination of other impurity elements that remain in the molten silicon phase as shown in Figure 4.

#### 3.1.3 Effect of the temperature on evaporation refining

Efficiency of the evaporation refining is determined by the difference of vapor pressures of impurity elements and that of molten silicon. It is found that the dependency of the elimination of the impurity elements by evaporation refining on temperature does not change significantly based on the systematic comparison of the vapor pressures of 23 impurity elements in the dilute concentration and that of the pure molten silicon at different temperatures (Supporting Information). Evaporation refining is possible to eliminate the impurity elements with higher vapor pressures than that of the molten silicon, such as phosphorus, zinc, lead, magnesium, and antimony. Meanwhile, a lower temperature is found to have slight advantages in the elimination of these impurity elements. For other impurity elements, however, evaporation refining process is not suitable.

### 3.2 Purification of silicon wafers from EoL PV panels by solvent refining

#### 3.2.1 Selection of the solvent metals for EoL silicon wafers refining

**Figure 7.**
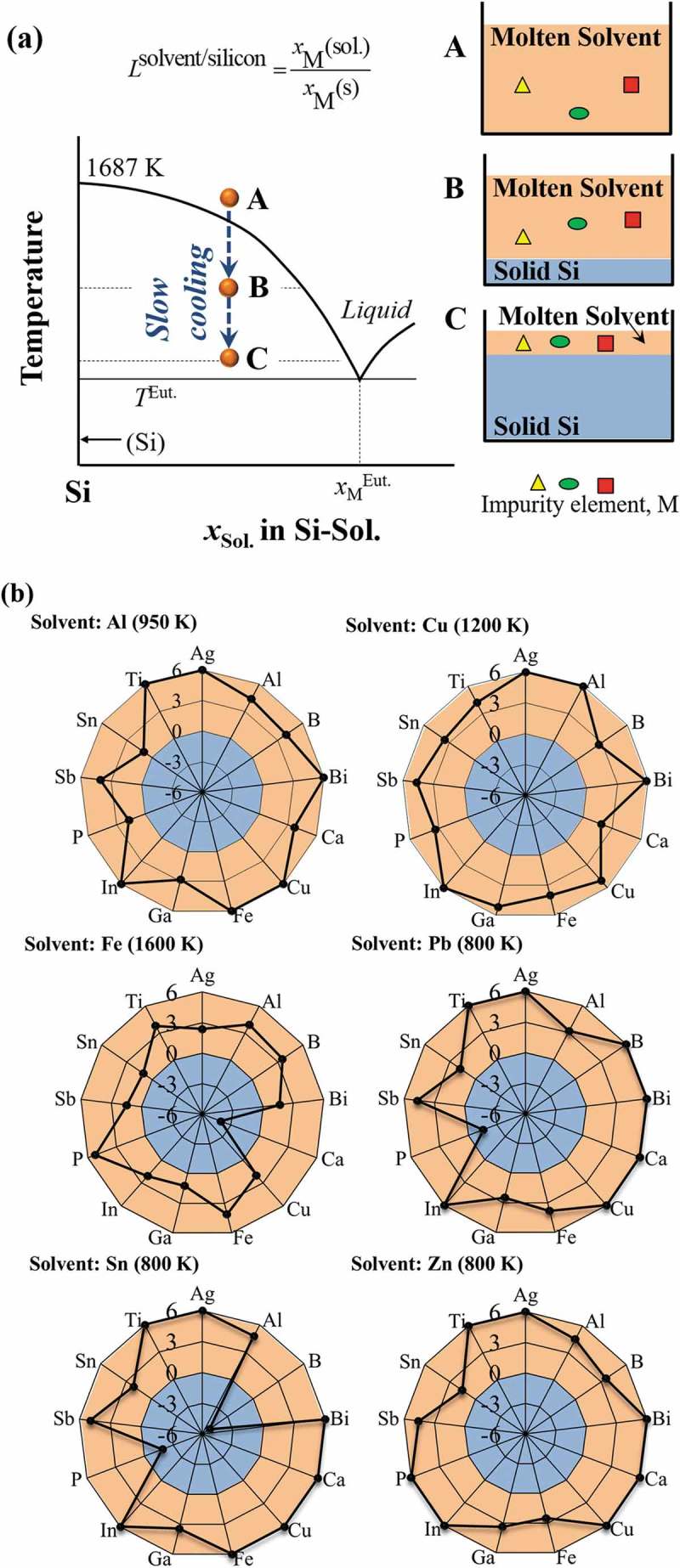
(a) Schematic solvent refining process based on the binary Si-Sol. phase diagram, and (b) the elimination limit of the typical impurity elements in silicon feedstocks by solvent refining process using aluminum, copper, iron, lead, tin, and zinc as the solvent metals, illustrated by using the logarithm of the distribution ratio of impurity element between solvent metal and silicon (log(*L* ^solvent/silicon^)).

Figure 7(a) schematically illustrates the solvent refining process of the EoL silicon wafers. By introducing solvent metal, silicon wafers containing impurity elements can be melted at temperature lower than its melting point (1687 K) as binary Si-Sol. alloy (step A). Silicon will be solidified and separated from the molten solvent phase by slowly cooling the binary melt, and impurity elements that have stronger affinity to the molten solvent phase will be eliminated from silicon (step B). Finally, silicon will be purified by proceeding the slow cooling process to remove the impurity elements (step C). The elimination of a given impurity element, M, by the solvent refining process is determined by its equilibrium distribution ratio between solid silicon and molten solvent metals (*L* ^solvent/silicon^).

A perfect metal for efficient solvent refining of EoL silicon wafers should have at least the following characteristics:

- significantly different density from the solid silicon to promote the ease of the separation of the liquid solvent metal from purified silicon;
- extremely low solubility in the solid silicon to avoid contamination of the solvent metal;
- low eutectic/peritectic temperature close to the silicon side in the binary silicon systems;
- a flat liquidus on the Si-rich side of the binary silicon system to efficiently solidify silicon and to reduce the silicon loose into the solvent metal, which is indicated as the ratio of the difference of the melting point of silicon (1687 K) and the eutectic temperature, to the eutectic composition;
- a much lower production cost comparing with that of high purity silicon.

14 metals with potential for use as solvent metals are compared in Table 1. Based on the prerequisite characteristics listed above, aluminum, copper, iron, lead, tin, and zinc were selected as the candidate solvent metals for EoL silicon wafers purification, and the removability of typical impurity elements using these solvent metals was investigated. Silver, cobalt, and gallium are too costly [50,51]. The eutectic temperatures for cobalt, chromium, iron, magnesium, manganese, nickel, and titanium are too high, whereas iron shows the advantages of extremely low cost and low solubility in solid silicon. Aluminum has advantages of low cost and low eutectic temperature, though its density is close to silicon, which make it difficult to separate liquid aluminum from solid silicon by the common gravity method and another method, such as acid leaching, may be required. Lead, tin, and zinc are preferred as the solvent metals, because all the binary systems have a eutectic point close to the solvent metal side, the density differences are suitable, and the cost is not as high as polysilicon.

**Table 1.** Comparison of metals with potential for use as solvent metals to refine EoL silicon wafers.

Candidate solvent metals	*ρ* _m_ (g/m^3^) ^1)^ [52]	Maximum Solubility in Si(s) (at. %)	Melting point (K)	*T*^Eut.^ (K) ^2)^	*x*_M_^Eut. 3)^	ΔT/*x*_M_^Eut.^(×10^−2^) ^4)^
Si	2.57	–	1687	–	–	–
Ag [53]	9.32	4 × 10^−4^ (1623 K)	1235	1072 (Eutectic)	0.89	6.9
Al [54]	2.38	0.016 ± 0.003 (1463 K)	933	850 (Eutectic)	0.878	9.5
Co [55]	7.75	2.6 × 10^−5^ (1543 K)	1768	1532 (Eutectic)	0.125	12.4
Cr [56]	6.28	8 × 10^−6^ (1578 K)	2133	1578 (Eutectic)	0.18	6.1
Cu [57]	8.00	0.002 (1473 K–1573 K)	1385	1075 (Eutectic)	0.699	8.8
Fe [58]	7.04	3.0 × 10^−5^ (1473)	1809	1480 (Eutectic)	0.265	7.8
Ga [59]	6.08	0.1 (1473 K)	303	303 (Eutectic)	0.99994	13.8
Mg [60]	1.58	Negligible	922	1219 (Eutectic)	0.47	10.0
Mn [61]	5.95	Negligible	1519	1423 (Eutectic)	0.321	8.2
Ni [62]	7.81	0.0014 (1573 K)	1728	1266 (Peritectic)	0.41	10.3
Pb [63]	10.66	Negligible	601	601 (Eutectic)	0.999996	10.9
Sn [64]	7.00	0.1 (1339K)	505	505 (Eutectic)	0.9999995	11.8
Ti [65]	4.11	Negligible	1943	1603 (Eutectic)	0.162	5.2
Zn [66]	6.57	0.00015 (1627 K)	693	692 (Eutectic)	0.99955	10.0

1) *ρ*_m_: the density of elements at melting point (g/m^3^).

2) *T* ^Eut.^: the eutectic temperature closest to silicon side in the binary Si-Sol. systems.

3) *x*_M_^Eut.^: the eutectic composition closest to silicon side in the binary Si-Sol. systems.

4) ΔT/*x*_M_^Eut.^: the ratio of the difference of the melting point of silicon (1687 K) and *T* ^Eut^ (K), to *x*_M_^Eut.^, which is used to indicate the shape of the liquidus.

#### 3.2.2 Purification of silicon wafers by solvent refining

The purification of the silicon wafers from EoL PV panels by solvent refining using aluminum, copper, iron, lead, tin, and zinc as the solvent metals was systematically investigated. Figure 7(b) shows the removability of 13 selected impurity elements by solvent refining process using six different solvent metals. The 13 selected elements include the typical dopant elements in c-Si PV panels (boron, phosphorus, antimony, and other Group IIIA and VA elements: bismuth, gallium, indium) [18], typical metal electrodes in PV panels (silver, copper, and tin) [10], as well as the typical impurity elements in MG-Si (iron, calcium, and titanium) [16]. In Figure 7(b), the elements that are distributed in the outer orange area can be thermodynamically removed into the solvent metal phase. If the calculated value of the logarithm of the distribution ratio of impurity element, log(*L* ^solvent/silicon^), is larger than 6, indicating an extremely strong distribution tendency into the solvent metal phase, the value of 6 will be set for such impurity element. The temperature of solvent refining process was set to 950 K, 1200 K, 1600 K, 800 K, 800 K, and 800 K for the processes that use aluminum, copper, iron, lead, tin, and zinc as the solvent metals according to the eutectic temperature, respectively.

The thermodynamic evaluations indicate that solvent metals of aluminum, copper, zinc, and iron can efficiently eliminate the typical impurity elements contained in the EoL c-Si PV panels, including the typical dopants and the metal electrodes. The solvent refining process using aluminum, copper, and zinc as solvent metals can efficiently eliminate all the considered 13 impurity elements. Elimination of calcium using iron as the solvent metal, elimination of phosphorus using lead as the solvent metal, and elimination of boron and phosphorus using tin as the solvent metal was found difficult. Considering the high likelihood of boron and phosphorus contamination as the mostly used dopants in PV panels, lead and tin are not suitable as the solvent metals for the refining of the silicon wafers during the recycling of the EoL PV panels. However, lead and tin might be wonderful solvent metals for the refining of MG-Si owing to the high distribution ratios of iron, calcium, and titanium into the solvent metals.

Among solvent metals of aluminum, copper, zinc, and iron, the removability of the dopant elements in c-Si PV panels using aluminum, copper, and zinc as the solvent metals is generally higher than that using iron as the solvent metal. Particularly, the removability of phosphorus using zinc, iron, and copper as the solvent metals is higher (zinc solvent: log(*L* ^solvent/silicon^) = 17.4; iron solvent: log(*L* ^solvent/silicon^) = 5.2; copper solvent: log(*L* ^solvent/silicon^) = 3.4), and the removability of boron using aluminum, zinc, and iron as the solvent metals is higher (aluminum solvent: log(*L* ^solvent/silicon^) = 4.0; zinc or iron solvent: log(*L* ^solvent/silicon^) = 3.6), respectively. Lead has the highest removability for boron (log(*L* ^solvent/silicon^) = 6.1), however, cannot eliminate another typical dopant element phosphorus (log(*L* ^solvent/silicon^) = −1.6). The evaluated distribution tendency of impurity elements between the molten solvent metal and solid silicon agree with the experimental ones, like that using aluminum as the solvent metal [67–69], despite the different temperatures used. The removability of impurity elements quantitatively illustrated in the radar charts is expected to be easily used for design of the solvent refining process of the silicon wafers during the EoL PV panels recycling.

Besides the thermodynamics, kinetic parameters also highly influence the efficiency of EoL silicon wafers refining. The oxidation refining and evaporation refining is carried out at higher temperature, in which condition the element diffusion through the melts of silicon and slag bulk, as well as the chemical reactions are generally fast. The possible rate-determining steps might be the mass transfer across the silicon melt and slag boundary layers for the oxidation refining [70], and the mass transfer across the silicon melt and gas boundary layers for the evaporation refining [71]. Increasing the refining temperature and improvement of the mixing condition by gas stirring were found beneficial for the impurity removal from silicon melts [70,71]. Comparing with oxidation refining and evaporation refining process, the solvent refining processes are generally carried out at lower temperature and the removal of impurity elements highly depends on the kinetic parameters [72]. At lower temperature, the diffusion coefficient of impurity elements in the melts is small [73]. Moreover, the viscosity of the melts becomes large during cooling, which will reduce the effect of convection. Both factors make the removal of the impurity elements from the growing solid silicon phase to the bulk of the alloy melts difficult at high cooling rate [73]. Moreover, though the solubility of the selected solvent metals in silicon is extremely small, high cooling rate might cause contamination of the solvent metals that is residual in the solidified silicon under nonequilibrium condition. Thus, control of the cooling rate, as well as promotion of the solute homogenization by methods such as electromagnetic stirring becomes crucial for the solvent refining process [72,73]. Consideration of these kinetic parameters is also important for design of the optimal EoL PV panels recycling process.

Consequently, the first choice to close the recycling loop of EoL c-Si PV panels is to purify the EoL silicon wafers using solvent refining process with aluminum, copper, and zinc as the solvent metals, in which process all the typical dopants and the metal electrodes can be efficiently eliminated. As the second choice, solvent refining process using iron as solvent metal can also work. Comparing with solvent refining process, re-melting process can also efficiently remove most of the dopant elements, while is insufficient to remove indium, gallium, and the attached metal electrodes. Particularly, re-melting process might be an optimal choice if sorting and pre-separation of the different types of silicon wafers and separation of the attached metal electrodes (silver, tin, and copper) are completed. A comprehensive management and recycling system considering the metallurgical criteria of the refining process of the EoL silicon wafers to face the coming boom of the disposed EoL PV panels is crucial to promote the recycling efficiency.

## 4. Conclusions

For a total of 42 impurity elements that are likely to be present in the collected EoL c-Si PV panels, their elimination limitation from silicon by three most typical metallurgical refining processes, oxidation refining, evaporation refining, and solvent refining, was quantitatively evaluate. The main conclusions of this study are as follows.

**Figure 8.**
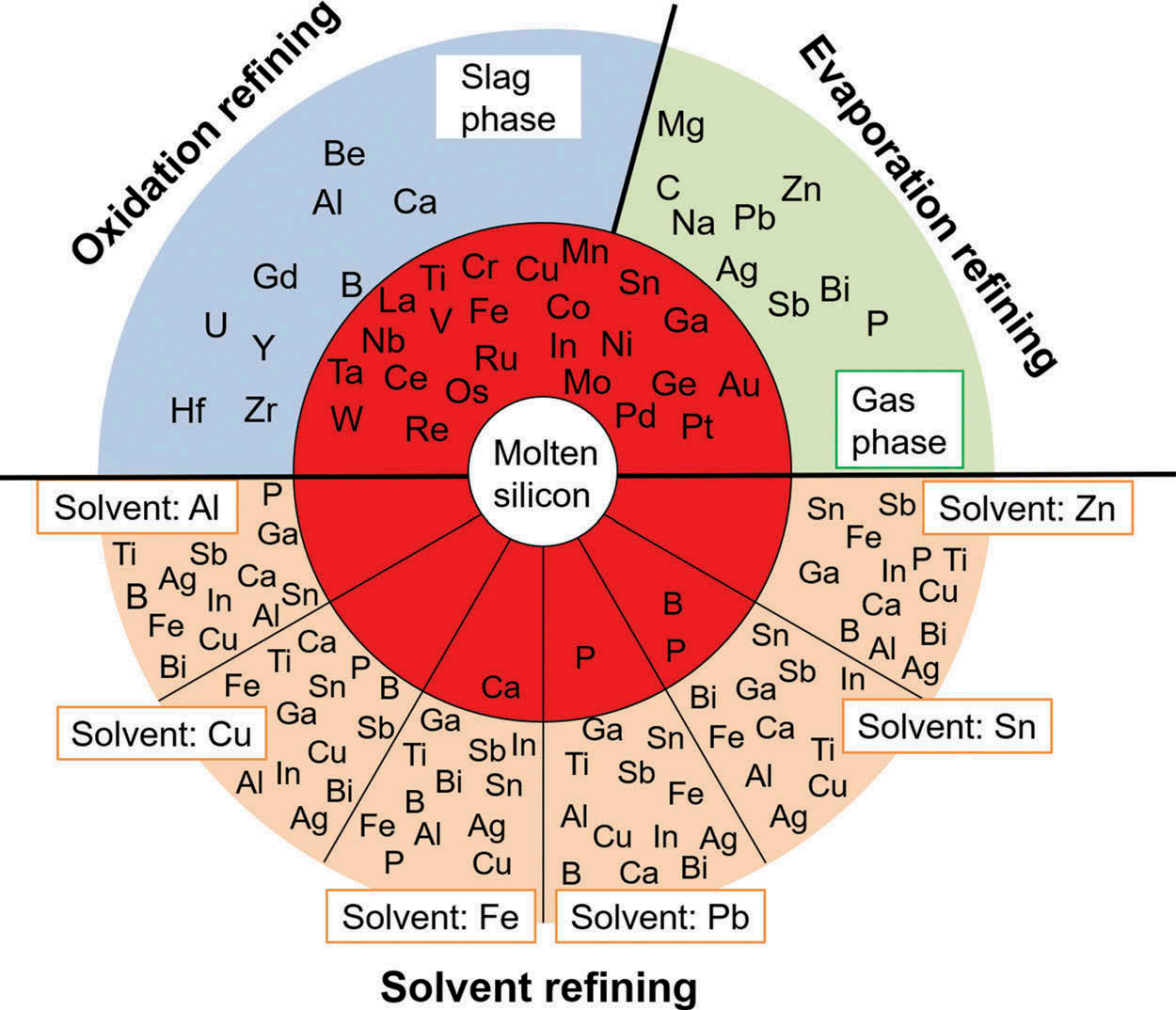
The thermodynamic criteria of the refining process of the EoL silicon wafer by oxidation refining, evaporation refining, and solvent refining with different solvent metals.

1. During the EoL silicon wafers remelting process with slag, removal of elements like aluminum, beryllium, calcium, gadolinium, hafnium, uranium, yttrium, and zirconium into the slag phase is possible. The removal of boron which have an almost equal distribution tendency into slag and the metal phases, by slag treatment might be possible if the suitable slag is used.
2. Antimony, bismuth, carbon, lead, magnesium, phosphorus, silver, sodium, and zinc can be removed into the vapor phase by evaporation. On the other hand, removal of the other considered 24 impurity elements by either oxidation or by evaporation during the re-melting process is difficult.
3. The effect of temperature on the elimination of impurity elements by either oxidation refining or evaporation refining is not remarkably large, while the removal of boron into the slag phase can be promoted at higher temperature.
4. The evaluated distribution ratios *L* ^slag/silicon^ agree well with the experimentally observed ones using different practical slag (within ±2 orders), and the effect of the slag composition on the removal of the impurity elements into slag phase is found to be insignificant.
5. Among the considered 14 metals with potential for use as solvent metals, aluminum, copper, and zinc are found efficient for refining of EoL silicon wafers by solvent refining process, in which process all the typical dopants and the metal electrodes can be efficiently eliminated. Particularly, purification of the phosphorus doped *n*-type PV panels using solvent metal zinc (log(*L* ^solvent/silicon^) = 17.4 for phosphorus) and purification of the boron doped *p*-type PV panels using solvent metal aluminum (log(*L* ^solvent/silicon^) = 4.0 for boron) are preferable.
6. A comprehensive management and recycling system considering the thermodynamic criteria of the refining process of the EoL silicon wafers, as summarized in Figure 8, to face the coming boom of the disposed EoL PV panels is crucial to promote the recycling efficiency.
