# Supplementary material for: Thermodynamic criteria of the end-of-life silicon wafers refining for closing the recycling loop of photovoltaic panels
Source: Sci Technol Adv Mater. 2019 Jul 10;20(1):813–25. doi: 10.1080/14686996.2019.1641429 (PMC6711135; doi:10.1080/14686996.2019.1641429)
Supplement: Supplemental Material [file TSTA_A_1641429_SM5609.docx]

**Supporting Information for**

**Thermodynamic criteria of the End-of-Life silicon wafers refining for closing the recycling loop of photovoltaic panels**

Xin Lu,^1,*^ Takahiro Miki,^1^ Osamu Takeda, ^1^ Hongmin Zhu, ^1^ Tetsuya Nagasaka^1^

^1^Graduate School of Engineering, Tohoku University, Miyagi 980-8579, Japan

Corresponding Author

*E-mail: [xin.lu.a5@tohoku.ac.jp](mailto:xin.lu.a5@tohoku.ac.jp); Tel & Fax: +81-22-795-7311.

**Number of pages: 36**

**Figures: 2**

**Tables: 10**

**References: 140**

# **S1.　Metallurgical refining processes of metallurgical-grade silicon (MG-Si)**

The conventional production and purification of solar grade silicon (SoG-Si) starts from carbothermic reduction of high-purity quartz (SiO_2_) above 1800 ºC using coke to produce the metallurgical-grade silicon (MG-Si) of approximately 98% purity. MG-Si is then purified to produce electronic-grade silicon (EG-Si) of extremely high purity (>11N) using a chemical gaseous purification technique known as the Siemens method.[1] Conventionally, the raw materials for SoG-Si are from off-grade products during the purification process of EG-Si. However, the supply of off-grade EG-Si materials for SoG-Si is facing an increasing risk owing to the improvement of the EG-Si fabrication technology and the promotion of the scrap recycling inside the EG-Si process scope. Further, the Siemens purification process is extremely complicated and energy intensive. With such concerns, lots of effort have been made to promote the SoG-Si supply from alternative resources.

In past decades, large efforts have been made to produce SoG-Si by direct refining MG-Si. [2–9] Particularly, the high-temperature refining process including (1) solvent refining [4,10] with different solvents such as aluminum [11,12], copper [13], iron [14–16], nickel [17], and other metals [18–20]; (2) oxidative refining using various slags [21–24]; (3) vacuum refining by electron beam melting, plasma melting and so on [25–27]; (4) directional solidification [28]; and (5) electro-refining [29,30] have been extensively focused on.

# **S2. The activity coefficients of impurity elements in molten silicon**

The Redlich-Kister-type polynomial is always employed to illustrate the excess Gibbs energy for solution mixing in the CALPHAD approach [31]. The interaction parameters for the binary system of Si-M system described by Redlich-Kister polynomial have been assessed in lots of literature studies, and the activity coefficients of the impurity elements can be calculated using the assessed interaction parameters. The activity coefficient of impurity element M in the binary Si-M system can be calculated by the following polynomials derived from Redlich-Kister polynomial.

| **$\begin{matrix} \text{R}T\ln\gamma_{\text{M}}={}^{0}\Omega_{\text{Si-M}}x_{\text{Si}}^{2}+{}^{1}\Omega_{\text{Si-M}}x_{\text{Si}}^{2}(4x_{\text{Si}}-3)+{}^{2}\Omega_{\text{Si-M}}x_{\text{Si}}^{2}(2x_{\text{Si}}-1)(6x_{\text{Si}}-5) \\ +{}^{3}\Omega_{\text{Si-M}}x_{\text{Si}}^{2}(2x_{\text{Si}}-1)^{2}(8x_{\text{Si}}-7) \end{matrix}$** | (S1) |
| --- | --- |

Since the concentration of impurity elements in the silicon is extremely low in most cases, using activity coefficients of M in the dilute solution,**$\gamma_{\text{M}}^{0}$**, is more correct. **$\gamma_{\text{M}}^{0}$** can be calculated much simpler at the limiting case of****:

| **$\text{R}T\ln\gamma_{\text{M}}^{0}=\sum_{p=0}^{3} {}^{p}\Omega_{\text{Si-M}}$** | (S2) |
| --- | --- |

where${}^{p}\Omega_{\text{Si-M}}$(*p*=0, 1, 2, 3) are the temperature-dependent binary interaction parameters and expressed as follows:

| ${}^{p}\Omega_{\text{Si-Mi}}={}^{p}\text{A}+{}^{p}\text{B}\cdot T+{}^{p}\text{C}\cdot\ln T+{}^{p}\text{D}\cdot T^{2}$ | (S3) |
| --- | --- |

where *^p^*A, *^p^*B, *^p^*C, and *^p^*D are constants

The calculated activity coefficients of impurity elements in silicon at 1773 K are listed in **Table S1**. The temperature-dependent binary interaction parameters, ${}^{p}\Omega_{\text{Si-M}}$ (*p*=0, 1, 2, 3), for the assessment of the liquid phase in Si-M binary system obtained in the literatures are shown in **Table S2**.

**Table S1.** The activity coefficient of impurity elements in infinite dilute liquid silicon solution at 1773 K in pure liquid standard state

| M | *γ*_M_(l) | M | *γ*_M_(l) |
| --- | --- | --- | --- |
| Ag | 3.28 | Mo | 2.35E-03 |
| Al | 0.50 | Na | 2.00E-02 |
| Au | 8.37E-02 | Nb | 1.46E-04 |
| B | 3.87 | Ni | 2.90E-03 |
| Be | 5.29 | Os | 5.08E-03 |
| Bi | 30.49 | P | 0.37 |
| C | 2.64 | Pb | 36.38 |
| Ca | 2.35E-03 | Pd | 1.32E-03 |
| Ce | 9.67E-10 | Pt | 1.54E-05 |
| Co | 7.16E-03 | Re | 5.12E-04 |
| Cr | 1.16E-02 | Ru | 0.15 |
| Cu | 0.32 | Sb | 5.09 |
| Fe | 2.55E-02 | Sn | 7.49 |
| Ga | 1.52 | Ta | 1.25E-03 |
| Gd | 7.33E-06 | Ti | 2.29E-04 |
| Ge | 1.50 | U | 2.16E-04 |
| Hf | 1.43E-05 | V | 4.73E-03 |
| In | 4.57 | W | 7.81E-03 |
| La | 7.95E-08 | Y | 2.12E-07 |
| Mg | 0.364 | Zn | 1.48 |
| Mn | 8.978E-03 | Zr | 2.67E-05 |

.

**Table S2.** The temperature-dependent binary interaction parameters (Redlich-Kister parameters) for the assessment of the liquid phase in Si-M binary systems.

| M | ${}^{0}\Omega_{\text{Si-M}}^{\text{Liq}}$ | ${}^{1}\Omega_{\text{Si-M}}^{\text{Liq}}$ | ${}^{2}\Omega_{\text{Si-M}}^{\text{Liq}}$ | ${}^{3}\Omega_{\text{Si-M}}^{\text{Liq}}$ | ${}^{4}\Omega_{\text{Si-M}}^{\text{Liq}}$ | Ref. |
| --- | --- | --- | --- | --- | --- | --- |
| Ag | 11052.0-0.09508T | 16066.4-5.31210T |  |  |  | Chevalier 1988 [32] |
| Al | -11340.1-1.23394T | 3530.9-1.35993T | 2265.4 |  |  | Gröbner 1996 [33] |
| Au | -24103.30-15.14T | 29375.28-1.11T | -13032.24 |  |  | Meng 2007 [34] |
| B | 2400+9.89T |  |  |  |  | Yoshikawa 2005 [35] |
| Be | -28151.67+27.75T | -4949.18 | 8455.14 |  |  | Pan 2005 [36] |
| Bi | 46370+2.26T |  |  |  |  | Olesinski 1985 [37] |
| C | 25645-6.38T |  |  |  |  | Gröbner 1996 [33] |
| Ca | -228147.9+61.7892T | 110482.3-73.61283T | 44663.2-6.95691T | -82284+56.03509T |  | Anglezio 1994 [38] |
| Ce | -99974.63-61.61T | 63423-90.35T |  |  |  | Gröbner 2004 [39] |
| Co | -183483.8+34.80023T | 3219.5+15.28341T | 34241.7 | -15579.7 |  | Zhang 2006 [40] |
| Cr | -128000+21.23883T | 50016.61-14.31913T |  |  |  | Chen 2009 [41] |
| Cu | -38763.5+12T | 52431.2-27.4571T | -29426.5+14.775T |  |  | Yan 2000 [42] |
| Fe | -164435+41.977T | 21.523T | 5220+5.726T | 28955-26.275T |  | Miettinen 1999[43] |
| Ga | 14900-4.9T |  |  |  |  | Olesinski 1985 [44] |
| Gd | -265000+60T | -60000+25T |  |  |  | Huang 2007 [45] |
| Ge | 6610-0.354T |  |  |  |  | Jung 2010 [46] |
| Hf | -177631+6.43T | 1830 |  |  |  | Zhao 2000 [47] |
| In | 45100-12.8T |  |  |  |  | Olesinski 1985 [48] |
| La | -249912.5-39.8T | -25123.5 | 83012.5 | 21695.6 |  | Zhou 2010 [49] |
| Mg | -73623.6+20.9297T | 40644.7-23.7571T | -37653.7+35.7315T | -80905.8+44.1741T | -12484.9 | Lüdecke 1986 [50] |
| Mn | -152854+32.125T | 38217+4.414 | 42606-16.74T | -32556 |  | Lee 2012 [51] |
| Mo | -158013.345+12T | -20000+15.015T | 39026.3+5.1567T | 2461.9-5.5087T |  | Guo 2012 [52] |
| Na | -58420+16.94T | -47005+9.99T |  |  |  | Hao 2012 [53] |
| Nb | -199000 | 18800 | 50000 |  |  | Geng 2009 [54] |
| Ni | -205000+33T | 102700-27T | 25000 | -117000+55T |  | Miettinen 2005 [55] |
| Os | -125865+31.56T | -27569+11.07T |  |  |  | Liu 2001[56] |
| P* | $\ln\gamma_{\text{P}}^{\text{o}}(\text{l})=0.197-\frac{2117}{T}$ | | | | | Yuan 2017 [57] |
| Pb | 66630-7.70T |  |  |  |  | Olesinski 1984 [58] |
| Pd | -253536.9+56.50T | 238372.9-86.77T | -127556.8+64.18T | -174993.5+71.85T | 178995.5-82.65T | Du 2006 [59] |
| Pt | -285834.40+32.71T | 110230.07-16.34T | 99818.56-27.74T | -111425.53+24.83T |  | Xu 2008 [60] |
| Re | -96700 | 45000 | -60000 |  |  | Shao 2001 [61] |
| Ru | -127858-27.62T | -59628+23.85T | 50985 |  |  | Liu 2001 [56] |
| Sb | 15463.82+4.81T |  |  |  |  | Wang 2011 [62] |
| Sn | 41468.97-10.94T | -30861.57+21.70T |  |  |  | Long 2012 [63] |
| Ta | -234317.343+44.55T | 77520.5861-11.68T |  |  |  | Drouelle 2013 [64] |
| Ti | -255852.17+21.874T | 25025.35-2.0023T | 83940.65-6.71526T |  |  | Seifert 1996 [65] |
| U | -185537+26.42 | -98478+53.79 | 47133-16.79T |  |  | Berche 2009 [66] |
| V | -190326.8+44.06T | -6265.4 | 39546.5 |  |  | Zhang 2008 [67] |
| W | -140000+35.1T | -20897.8 | -14660.85 |  |  | Li 2013 [68] |
| Y | -262885.3+87.63T | 5429.8-1.81T | -76199.8-25.40T |  |  | Ran 1989 [69] |
| Zn | 9692.3-1.29635T | -7943.3+3.57871T |  |  |  | Mey 1986 [70] |
| Zr | -200000+15.2454T | 10T |  |  |  | Chen 2009 [71] |

* The activity coefficient of phosphorus in infinite dilute silicon melts was evaluated and described by the shown equation.

# **S3. The activity coefficients of impurity elements in solid silicon**

The temperature-dependent binary interaction parameters for the assessment of the silicon-rich solid solution phase (diamond phase) in Si-M binary system obtained in the literatures are shown in **Table S3**. The activity coefficient of the considered impurity elements in the dilute solid silicon can be calculated using the similar method as **Equation S1** shown above.

**Table S3** The temperature-dependent binary interaction parameters (Redlich-Kister parameters) for the assessment of the silicon-rich solid solution phase in Si-M binary systems.

| M | ${}^{0}\Omega_{\text{Si-M}}^{\text{Solid}}$* | | Ref. |
| --- | --- | --- | --- |
|  | A | B |  |
| Ag | 280000 | -71.5 | Yoshikawa, et al. 2010 [72] |
| Al | 93200 | -14.5 | Yoshikawa, et al. 2010 [72] |
| B | 80800 | -26.3 | Yoshikawa, et al. 2010 [72] |
| Bi | 251000 | -74.7 | Yoshikawa, et al. 2010 [72] |
| Ca | 80 |  | Anglezio, 1994 [38]  Yoshikawa, et al. 2010 [72] |
| Cu | 162000 | -39.6 | Yoshikawa, et al. 2010 [72] |
| Fe | 137650 | -8.1 | Tang and Tangstad, 2012 [73] |
| Ga | 67700 | -3.81 | Yoshikawa, et al. 2010 [72] |
| In | 207000 | -53.8 | Yoshikawa, et al. 2010 [72] |
| P | -40498.5 | 21.8079 | Liang and Fetzer, 2014. (model I) [74] |
| Sb | 67795.81 |  | Wang, et al. 2011 [62] |
| Sn | 18725.62 | 22.2896 | Long, et al. 2012 [63] |
| Ti | 133000 | -41.2 | Yoshikawa, et al. 2010 [72] |

*

# **S4. The activity coefficients of impurity elements in molten solvent metals: aluminum, copper, iron, lead, tin, and zinc.**

The calculated activity coefficients of the impurity elements in different solvent metals and the used literature interaction parameters for the liquid Sol.-M (Sol.=Al, Cu, Fe, Pb, Sn, Zn) systems are listed in **Table S4**, while the activity coefficients were set as unity as the first approximation for some systems where the assessment results were unavailable. The temperature-dependent binary interaction parameters for the assessment of the liquid phase in Sol.-M (Sol.=Al, Cu, Fe, Pb, Sn, Zn) binary system obtained in the literatures are shown in **Table S5 − Table S10**. The activity coefficient of the considered impurity elements in the liquid solvent metals can be calculated using the similar method as **Equation S1** shown above.

**Table S4.** The activity coefficients of impurity elements in different solvent metals.

| M | *γ*_M_(l) in Aluminium  (905 K) | *γ*_M_(l) in copper  (1200 K) | *γ*_M_(l) in iron  (1600 K) |
| --- | --- | --- | --- |
| Ag | 0.011 | 3.15 | 860.8 |
| Al | 1.00 | 0.00030 | 0.012 |
| B | 5.31 | 3.70 | 0.017 |
| Bi | 1.01 | 4.37 | 82.26 |
| Ca | 0.00025 | 0.014 | 8421 |
| Cu | 0.011 | 1.00 | 13.94 |
| Fe | 0.00011 | 58.05 | 1.00 |
| Ga | 1.10 | 0.00075 | 1.00 |
| In | 22.22 | 0.042 | 36.82 |
| P | 0.0014 | 0.000082 | 0.0000032 |
| Sb | 0.41 | 0.011 | 1.75 |
| Sn | 7.42 | 0.0099 | 1.73 |
| Ti | 0.00042 | 0.48 | 0.029 |
|  |  |  |  |
| M | *γ*_M_(l) in lead  (800 K) | *γ*_M_(l) in tin  (800 K) | *γ*_M_(l) in zinc  (800 K) |
| Ag | 3.63 | 0.59 | 0.75 |
| Al | 177.30 | 4.82 | 8.09 |
| B | 1.00 | 1.66×10^11^ | 30.14 |
| Bi | 1.00 | 1.37 | 19.24 |
| Ca | 8.74×10^-8^ | 1.85×10^-8^ | 5.56×10^-8^ |
| Cu | 1.00 | 0.24 | 0.026 |
| Fe | 180950.88 | 98.97 | 3.64×10^6^ |
| Ga | 14.31 | 1.56 | 1,76 |
| In | 1.00 | 0.87 | 5.47 |
| P | 1.00 | 1.96 | 1.20×10^-19^ |
| Sb | 0.75 | 0.41 | 1.03 |
| Sn | 2.16 | 1.00 | 3.72 |
| Ti | 1.00 | 0.00021 | 0.087 |

**Table S5.** The temperature-dependent binary interaction parameters (Redlich-Kister parameters) for the assessment of the liquid phase in the Al-M binary systems.

| M | ${}^{0}\Omega_{\text{Al-M}}^{\text{Liq}}$ | ${}^{1}\Omega_{\text{Al-M}}^{\text{Liq}}$ | ${}^{2}\Omega_{\text{Al-M}}^{\text{Liq}}$ | ${}^{3}\Omega_{\text{Al-M}}^{\text{Liq}}$ | Ref. |
| --- | --- | --- | --- | --- | --- |
| Ag | -15300-8.8T | -25400+4.3T | 910+0.2T | 8500 | Lim 1995 [75] |
| B | -927.53-5.11T | 31961.6 | -12992 |  | Mirkovic 2004 [76] |
| Bi | 75.35-0.053T | 20.42-0.0037T |  |  | Kim 2006[77] |
| Ca | -89545+26.37T | -21847+11.77T | 4780+5.03T |  | Ozturk 2005 [78] |
| Cu | -67094+8.56T | 32148-7.12T | 5915-5.89T | -7290+5.5T | Liang 2015 [79] |
| Fe | -91976.5+22.13T | -5672.6+4.87T | 121.9 |  | Jacobs 2009 [80] |
| Ga | 2613.3-2.95T | 692.4-0.092T | 319.5 |  | Watson 1992 [81] |
| In | -10267+85.65T-0.055T^2^ | 8787-12.02T+0.0066T^2^ |  |  | Kaban 2010 [82] |
| P | -51769.9 |  |  |  | Liang 2013 [83] |
| Sb | -13328-5.10T | 10748+0.34T |  |  | Balakumar 2005 [84] |
| Sn | 16329.85-4.98T | 4111.97-1.15T | 1765.43-0.57T |  | COST 1998 [85] |
| Ti | -118048+41.97T | -23613+19.70T | 34757-13.84T |  | Witusiewicz 2008[86] |

**Table S6.** The temperature-dependent binary interaction parameters (Redlich-Kister parameters) for the assessment of the liquid phase in the Cu-M binary systems.

| M | ${}^{0}\Omega_{\text{Cu-M}}^{\text{Liq}}$ | ${}^{1}\Omega_{\text{Cu-M}}^{\text{Liq}}$ | ${}^{2}\Omega_{\text{Cu-M}}^{\text{Liq}}$ | ${}^{3}\Omega_{\text{Cu-M}}^{\text{Liq}}$ | ${}^{4}\Omega_{\text{Cu-M}}^{\text{Liq}}$ | Ref. |
| --- | --- | --- | --- | --- | --- | --- |
| Ag | 16914.95-14.77T+1.55TlnT | -1963.30+0.86T |  |  |  | He 2006 [87] |
| Al | -67094+8.56T | -32148+7.12T | 5915-5.89T | 7290-5.5T |  | Liang 2015 [79] |
| B | -3156.2 | -14390.26+9.98T | 42620-20T |  |  | Wang 2009 [88] |
| Bi | 23844.75-9.84T | 1260.32+1.19T |  |  |  | Teppo, 1990 [89] |
| Ca | -27967 | -9738 | -4994 |  |  | Risold 1996 [90] |
| Fe | 35625.8-2.19T | -1529.8+1.15T | 12714.4-5.19T | 1177.1 |  | Chen 1995 [91] |
| Ga | -58110.5+154.54T-18.38TlnT | -22884.7+1.92T | -11256.9 |  |  | Li 2008 [92] |
| In | -41564.8+238.62T-19.83TlnT | -76057.8+371.31T-44.99TlnT | -42076.5+192.40T-23.23TlnT |  |  | Liu 2002 [93] |
| P | -180379+101.07T | 39496-91.51T | 35583 |  |  | Noda 2009 [94] |
| Sb | -16154.82+24.00T-4.03TlnT | -35130.8+50.33T-5.23TlnT | -29263.28+15.32T | -2300.89 | 8873.94 | Liu 2000 [95] |
| Sn | -9935.17-5.16T | -21571.21+4.84T | -11005.75-2.61T |  |  | Li 2013 [96] |
| Ti | -19330+7.65T |  | 9382-5.45T |  |  | Kumar 1996[97] |

**Table S7.** The temperature-dependent binary interaction parameters (Redlich-Kister parameters) for the assessment of the liquid phase in the Fe-M binary systems.

| M | ${}^{0}\Omega_{\text{Fe-M}}^{\text{Liq}}$ | ${}^{1}\Omega_{\text{Fe-M}}^{\text{Liq}}$ | ${}^{2}\Omega_{\text{Fe-M}}^{\text{Liq}}$ | ${}^{3}\Omega_{\text{Fe-M}}^{\text{Liq}}$ | Ref. |
| --- | --- | --- | --- | --- | --- |
| Ag | 81089 | 8806 |  |  | Swartzendruber 1984 [98] |
| Al | -91976.5+22.13T | 5672.6-4.87T | 121.9 |  | Jacobs 2009 [80] |
| B | -126220+29.44T | -8390 | 33538 |  | Rompaey 2002 [99] |
| Bi | 62327.74-4.39T | 3362.48 |  |  | Boa 2006 [100] |
| Ca | 120233 |  |  |  | Anglezio 1994 [38] |
| Cu | 35625.8-2.19T | 1529.8-1.15T | 12714.4-5.19T | -1177.1 | Chen 1995 [91] |
| In | 52838.5-5.54T | 3999.9 |  |  | Ohno 2009 [101] |
| P | -266000+41.3T | 96900-40.84T |  |  | Ohtani 2006 [102] |
| Sb | -20094.45+16.48T | -17411.15+11.60T |  |  | Boa 2006 [100] |
| Sn | 108744.39-441.227T+52.18TlnT | -8992.66+2.03T | -9855.25+0.98T | 2552.59 | Huang 2010 [103] |
| Ti | -74300+17.84T | 8299.85-6.10T |  |  | Bo 2012 [104] |

**Table S8.** The temperature-dependent binary interaction parameters (Redlich-Kister parameters) for the assessment of the liquid phase in the Pb-M binary systems.

| M | ${}^{0}\Omega_{\text{Pb-M}}^{\text{Liq}}$ | ${}^{1}\Omega_{\text{Pb-M}}^{\text{Liq}}$ | ${}^{2}\Omega_{\text{Pb-M}}^{\text{Liq}}$ | Ref. |
| --- | --- | --- | --- | --- |
| Ag | 13330.32-6.21T | 1449.03+1.06T | -2089.13 | Lee 1994 [105] |
| Al | 47933.60-10.72T | -14407.33+6.65T | 4742.36-0.72T | Yu 1996 [106] |
| Ca | -104625.76-4.35T |  |  | Idbenali 2008 [107] |
| Fe | 110114.85-9.11T | 27699.55-6.74T |  | Vaajamo 2011 [108] |
| Ga | 13658.15+2.96T | -1420.89+1.39T | 5214.18-4.04T | Mathon 1996 [109] |
| Sb | -212.66-2.00T | -88.23 |  | Lee 1994 [105] |
| Sn | 6200-0.42T | 790-1.91T |  | Ohtani 1995 [110] |

**Table S9.** The temperature-dependent binary interaction parameters (Redlich-Kister parameters) for the assessment of the liquid phase in the Sn-M binary systems.

| M | ${}^{0}\Omega_{\text{Sn-M}}^{\text{Liq}}$ | ${}^{1}\Omega_{\text{Sn-M}}^{\text{Liq}}$ | ${}^{2}\Omega_{\text{Sn-M}}^{\text{Liq}}$ | ${}^{3}\Omega_{\text{Sn-M}}^{\text{Liq}}$ | Ref. |
| --- | --- | --- | --- | --- | --- |
| Ag | -399.49-31.42T+3.08TlnT | 18150.65-5.88T | -12009.03+5.18T |  | Gierlotka 2012 [111] |
| Al | 16329.85-4.98T | -4111.97+1.15T | 1765.43-0.57T |  | COST 507 1998 [85] |
| B | $RT\ln\gamma_{\text{B}}^{\text{o}}(\text{l})=188000(\pm6700)-20.2(\pm3.7)T$ | | | | Ma 2012 [112] |
| Bi | 446.6+0.88T | 1.21T |  |  | Lee 1996 [113] |
| Ca | -110358+3.18T | -10605 |  |  | Ohno 2006 [114] |
| Cu | -21571.21+4.84T | 11005.75+2.61T |  |  | Li 2013 [96] |
| Fe | 108744.39-441.227T+52.18TlnT | 8992.66-2.03T | -9855.25+0.98T | -2552.59 | Huang, 2010 [103] |
| Ga | 3369.7+0.039T | -528.9+0.11T |  |  | Anderson 1992[115] |
| In | -711-1.69T | 64+1.36T |  |  | Lee 1996 [113] |
| P | 16690-16.90T | 9696 | -8400 |  | Miettinen 2001 [116] |
| Sb | -5695.1-1.71T | -782.6 | 1840.9 |  | ManasijevićŽ 2008 [117] |
| Ti | -91598.90-0.94T | 45682.64-12.10T |  |  | Yin 2007 [118] |

**Table S10.** The temperature-dependent binary interaction parameters (Redlich-Kister parameters) for the assessment of the liquid phase in the Zn-M binary systems.

| M | ${}^{0}\Omega_{\text{Zn-M}}^{\text{Liq}}$ | ${}^{1}\Omega_{\text{Zn-M}}^{\text{Liq}}$ | ${}^{2}\Omega_{\text{Zn-M}}^{\text{Liq}}$ | ${}^{3}\Omega_{\text{Zn-M}}^{\text{Liq}}$ | Ref. |
| --- | --- | --- | --- | --- | --- |
| Ag | -27400+5.88 | 5500 |  |  | Gomez-Acebo 1998 [119] |
| Al | 10288+3.04T | 810+0.47T |  |  | Chen 1993 [120] |
| B | 91314.2-66.68T |  |  |  | Chen 2013 [121] |
| Bi | 17731-8.08T+0.044TlnT | 1988+3.50T+0.16TlnT | 3104+0.066T-0.16TlnT | 10934+11.30T+0.29TlnT | Djaballah 2005 [122] |
| Ca | -65435+10.62T | -43288+12.98T | -49709+25.55T |  | Brubaker 2001 [123] |
| Cu | -89687+369.49T-44.58TlnT | 4756-2.20T | 10513-6.78T |  | Wang 2011 [124] |
| Fe | 58088-23.67T | 92219-55.58T | 13570 |  | Nakano 2005 [125] |
| Ga | 3898.25+26.09T-4.09TlnT | -874.29 |  |  | Mathon 2000 [126] |
| In | 12401-4.45T | 3186-1.78T | 679 |  | Lee 1996 [127] |
| P | -264052.22exp(0.0001447T) | 13108.03exp(-0.00084T) |  |  | Liu 2016 [128] |
| Sb | -11951-1.12T | 3325.6-1.00T | 29432-23.63T |  | Li 2007 [129] |
| Sn | 12558-8.70T | 5623-4.20T | 4149-4.09T |  | Lee 1996 [127] |
| Ti | -30000+17.2T |  |  |  | Doi 2006 [130] |

# **S****5.** **Effect of the temperature on evaporation refining**

Efficiency of the evaporation refining is determined by the difference of vapor pressures of impurity elements and that of molten silicon. Comparison of the equilibrium vapor pressures of phosphorus gas in forms of P (g), P_2_(g), and P_4_ (g) over pure phosphorus was shown in **Figure S1**. It was noticed that the dominant vapor of phosphorus is P_4_ (g) under the equilibrium condition at the considered temperature range.

**Figure S2** systematically compares the vapor pressures of 23 impurity elements in the dilute concentration and that of the pure molten silicon at different temperatures. The dependency of the elimination of the impurity elements by evaporation refining on temperature does not change significantly, though the vapor pressure of the impurity element generally increases as the temperature increase. Some of the impurity elements, including phosphorus, zinc, lead, magnesium, and antimony, have higher vapor pressures, even in dilute concentration than that of pure silicon. Evaporation refining is suitable to eliminate these impurity elements. Meanwhile, a lower temperature is found to have slight advantages in the elimination of these impurity elements. Since the vapor pressure of silver is approximately the same as that of pure silicon, elimination of silver by evaporation refining can only work when some loss of silicon through evaporation is acceptable. For other impurity elements, however, evaporation refining process is not suitable even at high temperatures. It is especially difficult to eliminate boron the typical dopants in PV cells, using the evaporation refining process.


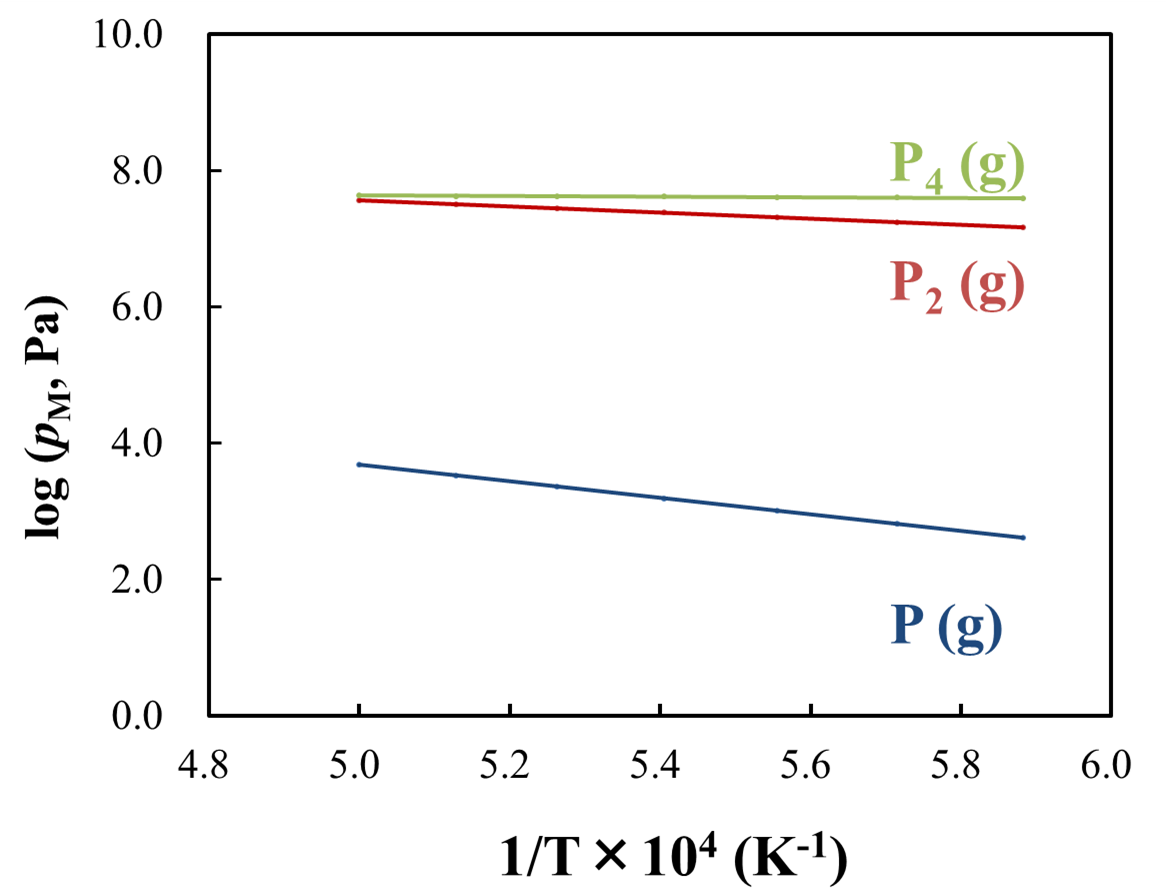


**Figure S1** Comparison of the equilibrium vapor pressures of phosphorus gas in forms of P (g), P_2_(g), and P_4_ (g) over pure phosphorus.

**
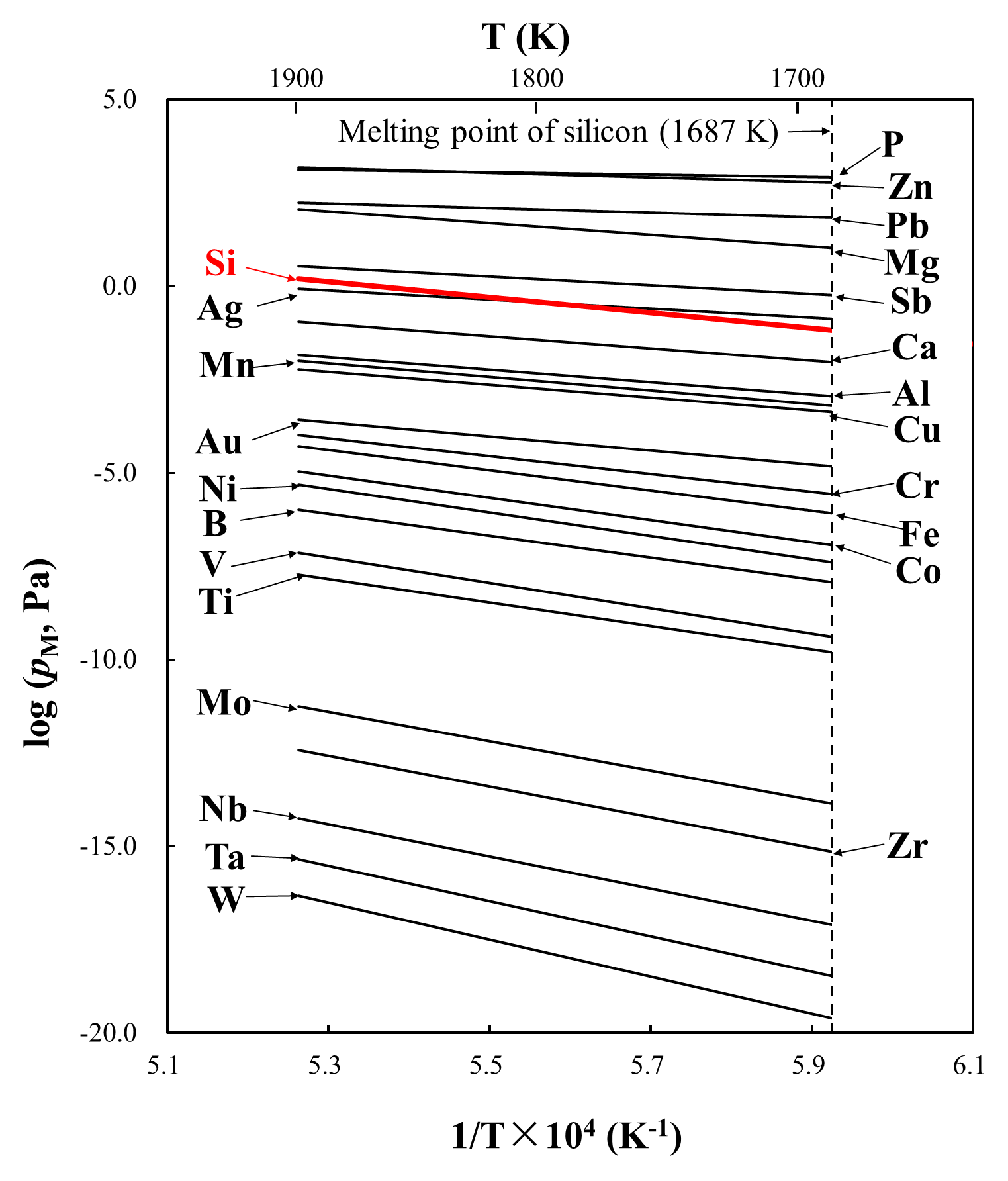
**

**Figure S2** Comparison of the vapor pressures of impurity elements in dilute concentration in silicon melt and that of pure molten silicon.

# **REFFERENCE**

[1] Takiguchi H and Morita K 2011 Global flow analysis of crystalline silicon *Crystalline silicon – properties and uses* vol 15 pp 329–44

[2] Safarian J, Tranell G and Tangstad M 2012 Processes for Upgrading Metallurgical Grade Silicon to Solar Grade Silicon *Energy Procedia* **20** 88–97

[3] Morita K and Miki T 2003 Thermodynamics of solar-grade-silicon refining *Intermetallics* **11** 1111–7

[4] Morita K and Yoshikawa T 2011 Thermodynamic evaluation of new metallurgical refining processes for SOG-silicon production *Trans. Nonferrous Met. Soc. China* **21** 685–90

[5] Braga A F B, Moreira S P, Zampieri P R, Bacchin J M G and Mei P R 2008 New processes for the production of solar-grade polycrystalline silicon: A review *Sol. Energy Mater. Sol. Cells* **92** 418–24

[6] Johnston M D, Khajavi L T, Li M, Sokhanvaran S and Barati M 2012 High-Temperature Refining of Metallurgical-Grade Silicon: A Review *JOM* **64** 935–45

[7] Yasuda K and H. Okabe T 2010 Production Processes of Solar Grade Silicon Based on Metallothermic Reduction *J. Jpn. Inst. Met.* **74** 1–9

[8] Yasuda K, Morita K and Okabe T H 2010 Production Processes of Solar Grade Silicon by Hydrogen Reduction and/or Thermal Decomposition *J. MMIJ* **126** 115–23

[9] Mukashev B N, Abdullin Kh A, Tamendarov M F, Turmagambetov T S, Beketov B A, Page M R and Kline D M 2009 A metallurgical route to produce upgraded silicon and monosilane *Sol. Energy Mater. Sol. Cells* **93** 1785–91

[10] Yoshikawa T and Morita K 2012 An Evolving Method for Solar-Grade Silicon Production: Solvent Refining *JOM* **64** 946–51

[11] Li J, Guo Z, Li J and Yu L 2015 Super Gravity Separation of Purified Si from Solvent Refining with the Al-Si Alloy System for Solar Grade Silicon *Silicon* **7** 239–46

[12] Li J and Guo Z 2014 Thermodynamic evaluation of segregation behaviors of metallic impurities in metallurgical grade silicon during AlSi solvent refining process *J. Cryst. Growth* **394** 18–23

[13] Mitrašinović A M and Utigard T A 2009 Refining Silicon for Solar Cell Application by Copper Alloying *Silicon* **1** 239–48

[14] Khajavi L T, Morita K, Yoshikawa T and Barati M 2015 Thermodynamic of boron distribution in solvent refining of silicon using ferrosilicon alloys *J. Alloys Compd.* **619** 634–8

[15] Esfahani S and Barati M 2011 Purification of metallurgical silicon using iron as impurity getter, part I: growth and separation of Si *Met. Mater. Int.* **17** 823–9

[16] Esfahani S and Barati M 2011 Purification of metallurgical silicon using iron as impurity getter, part II: extent of silicon purification *Met. Mater. Int.* **17** 1009–15

[17] Yin Z, Oliazadeh A, Esfahani S, Johnston M and Barati M 2011 Solvent refining of silicon using nickel as impurity getter *Can. Metall. Q.* **50** 166–72

[18] Ma X, Yoshikawa T and Morita K 2014 Purification of metallurgical grade Si combining Si–Sn solvent refining with slag treatment *Sep. Purif. Technol.* **125** 264–8

[19] Hu L, Wang Z, Gong X, Guo Z and Zhang H 2013 Purification of metallurgical-grade silicon by Sn–Si refining system with calcium addition *Sep. Purif. Technol.* **118** 699–703

[20] Li J, Ban B, Li Y, Bai X, Zhang T and Chen J 2017 Removal of Impurities from Metallurgical Grade Silicon During Ga-Si Solvent Refining *Silicon* **9** 77–83

[21] Johnston M D and Barati M 2010 Distribution of impurity elements in slag–silicon equilibria for oxidative refining of metallurgical silicon for solar cell applications *Sol. Energy Mater. Sol. Cells* **94** 2085–90

[22] Næss M K, Kero I, Tranell G, Tang K and Tveit H 2014 Element Distribution in Silicon Refining: Thermodynamic Model and Industrial Measurements *JOM* **66** 2343–54

[23] Jung I-H and Zhang Y 2012 Thermodynamic Calculations for the Dephosphorization of Silicon Using Molten Slag *JOM* **64** 973–81

[24] Olsen J E, Kero I T, Engh T A and Tranell G 2017 Model of Silicon Refining During Tapping: Removal of Ca, Al, and Other Selected Element Groups *Metall. Mater. Trans. B* **48** 870–7

[25] Sasaki H, Kobashi Y, Nagai T and Maeda M 2013 Application of Electron Beam Melting to the Removal of Phosphorus from Silicon: Toward Production of Solar-Grade Silicon by Metallurgical Processes *Adv. Mater. Sci. Eng.* **2013** 1–8

[26] Zheng S-S, Abel Engh T, Tangstad M and Luo X-T 2011 Separation of Phosphorus from silicon by induction vacuum refining *Sep. Purif. Technol.* **82** 128–37

[27] Safarian J and Tangstad M 2012 Vacuum Refining of Molten Silicon *Metall. Mater. Trans. B* **43** 1427–45

[28] Martorano M A, Neto J B F, Oliveira T S and Tsubaki T O 2011 Refining of metallurgical silicon by directional solidification *Mater. Sci. Eng. B* **176** 217–26

[29] Cai J, Luo X, Lu C, Haarberg G M, Laurent A, Kongstein O E and Wang S 2012 Purification of metallurgical grade silicon by electrorefining in molten salts *Trans. Nonferrous Met. Soc. China* **22** 3103–7

[30] Cai J, Luo X, Haarberg G M, Kongstein O E and Wang S 2012 Electrorefining of metallurgical grade silicon in molten CaCl2 based salts *J. Electrochem. Soc.* **159** D155–8

[31] Lukas H L, Fries S G and Sundman B 2007 *Computational Thermodynamics: The Calphad Method* (New York, USA: Cambridge University Press)

[32] Chevalier P-Y 1988 Thermodynamic evalution of the Ag-Si system *Thermochim. Acta* **130** 33–41

[33] Gröbner J, Lukas H L and Aldinger F 1996 Thermodynamic calculation of the ternary system Al-Si-C *CALPHAD* **20** 247–54

[34] Meng F G, Liu H S, Liu L B and Jin Z P 2007 Thermodynamic description of the Au-Si-Sn system *J. Alloys Compd.* **431** 292–7

[35] Yoshikawa T and Morita K 2005 Thermodynamic property of B in molten Si and phase relations in the Si–Al–B system *Mater. Trans.* **46** 1335–1340

[36] Pan Z, Du Y and Huang B Experimental investigation and thermodynamic calculation in the Al-Be-Si ternary system *Z. Für Met.* **96** 1301–7

[37] Olesinski R W and Abbaschian G J 1985 The Bi- Si (Bismuth-Silicon) system *Bull. Alloy Phase Diagr.* **6** 359–361

[38] Anglezio J C, Servant C and Ansara I 1994 Contribution to the experimental and thermodynamic assessment of the Al-Ca-Fe-Si system -I. Al-Ca-Fe, Al-Ca-Si, Al-Fe-Si and Ca-Fe-Si systems *Calphad* **18** 273–309

[39] Gröbner J, Mirković D and Schmid-Fetzer R 2004 Thermodynamic aspects of the constitution, grain refining, and solidification enthalpies of Al-Ce-Si alloys *Metall. Mater. Trans. A* **35** 3349–62

[40] Zhang L, Du Y, Xu H and Pan Z 2006 Experimental investigation and thermodynamic description of the Co–Si system *Calphad* **30** 470–81

[41] Chen H, Du Y and Schuster J C 2009 On the melting of Cr5Si3 and update of the thermodynamic description of Cr–Si *Calphad* **33** 211–4

[42] Yan X and Chang Y A 2000 A thermodynamic analysis of the Cu–Si system *J. Alloys Compd.* **308** 221–9

[43] Miettinen J 1999 Thermodynamic description of solution phases of systems Fe-Cr-Si and Fe-Ni-Si with low silicon contents and with application to stainless steels *Calphad* **23** 249–62

[44] Olesinski R W, Kanani N and Abbaschian G J 1985 The Ga−Si (Gallium-Silicon) system *Bull. Alloy Phase Diagr.* **6** 362–4

[45] Huang M, Schlagel D L, Schmidt F A and Lograsso T A 2007 Experimental investigation and thermodynamic modeling of the Gd–Si system *J. Alloys Compd.* **441** 94–100

[46] Jung I-H and Kim J 2010 Thermodynamic modeling of the Mg–Ge–Si, Mg–Ge–Sn, Mg–Pb–Si and Mg–Pb–Sn systems *J. Alloys Compd.* **494** 137–47

[47] Zhao J-C, Bewlay B P, Jackson M R and Chen Q 2000 Hf-Si binary phase diagram determination and thermodynamic modeling *J. Phase Equilibria* **21** 40–5

[48] Olesinski R W, Kanani N and Abbaschian G J 1985 The In−Si (Indium-Silicon) system *Bull. Alloy Phase Diagr.* **6** 128–30

[49] Zhou S, Liu L, Yuan X, Zheng F and Jin Z 2010 Thermodynamic assessment of La–Si and Mg–La–Si systems *J. Alloys Compd.* **490** 253–9

[50] Lüdecke D 1986 Phase diagram and thermochemistry of the Al-Mg-Si system *Z. Für Met.* **77** 278–83

[51] Lee Y E 2012 A Thermodynamic Assessment of Liquid Mn-Si alloy *ISIJ Int.* **52** 1539–43

[52] Guo C, Li C, Masset P J and Du Z 2012 A thermodynamic description of the Al–Mo–Si system *Calphad* **36** 100–9

[53] Hao D, Bu M, Wang Y, Tang Y, Gao Q, Wang M, Hu B and Du Y 2012 Thermodynamic modeling of the Na-X (X = Si, Ag, Cu, Cr) systems *J. Min. Metall. Sect. B Metall.* **48** 273–82

[54] Geng T, Li C, Bao J, Zhao X, Du Z and Guo C 2009 Thermodynamic assessment of the Nb–Si–Ti system *Intermetallics* **17** 343–57

[55] Miettinen J 2005 Thermodynamic description of the Cu–Ni–Si system in the copper-rich corner above 700 ∘C *Calphad* **29** 212–21

[56] Liu Y Q, Shao G and Homewood K P 2001 Thermodynamic assessment of the Ru–Si and Os–Si systems *J. Alloys Compd.* **320** 72–9

[57] Yan W, Yang Y, Chen W, Barati M and McLean A 2017 Thermodynamic assessment of Si-P and Si-Fe-P alloys for solar grade silicon refining via vacuum levitation *Vacuum* **135** 101–8

[58] Olesinski R W and Abbaschian G J 1984 The Pb−Si (Lead−Silicon) system *Bull. Alloy Phase Diagr.* **5** 271–3

[59] Du Z, Guo C, Yang X and Liu T 2006 A Thermodynamic description of the Pd–Si–C system *Intermetallics* **14** 560–9

[60] Xu L L, Wang J, Liu H S and Jin Z P 2008 Thermodynamic assessment of the Pt–Si binary system *Calphad* **32** 101–5

[61] Shao G 2001 Thermodynamic analysis of the Re–Si system *Intermetallics* **9** 1063–8

[62] Wang J, Liu Y J, Liu L B, Zhou H Y and Jin Z P 2011 Thermodynamic modeling of the Au–Sb–Si ternary system *J. Alloys Compd.* **509** 3057–64

[63] Long Z, Yin F, Liu Y, Wang J, Liu H and Jin Z 2012 Thermodynamic Description of the Ru-(Si,Ge)-Sn Ternary Systems *J. Phase Equilibria Diffus.* **33** 97–105

[64] Drouelle I and Servant C 2013 Thermodynamic assessment of the Si–Ta system *J. Alloys Compd.* **551** 293–9

[65] Seifert H J, Lukas H L and Petzow G 1996 Thermodynamic optimization of the Ti-Si system *Z. Für Met.* **87** 2–13

[66] Berche A, Rado C, Rapaud O, Guéneau C and Rogez J 2009 Thermodynamic study of the U–Si system *J. Nucl. Mater.* **389** 101–7

[67] Zhang C, Du Y, Xiong W, Xu H, Nash P, Ouyang Y and Hu R 2008 Thermodynamic modeling of the V-Si system supported by key experiments *Calphad* **32** 320–5

[68] Li Y, Li C, Du Z and Guo C 2013 Thermodynamic optimization of the Nb–Si–W ternary system *Calphad* **43** 112–23

[69] Ran Q, Lukas H L, Effenberg G and Petzow G 1989 A thermodynamic assessment of the Y-Si system *Z. Für Met.* **80** 402–5

[70] Sabine an Mey and Hack K 1986 A thermochemical evaluation of the Si-Zn, Al-Si and Al-Si-Zn system *Z. Für Met.* **77** 454–9

[71] Chen H M, Zheng F, Liu H S, Liu L B and Jin Z P 2009 Thermodynamic assessment of B–Zr and Si–Zr binary systems *J. Alloys Compd.* **468** 209–16

[72] Yoshikawa T, Morita K, Kawanishi S and Tanaka T 2010 Thermodynamics of impurity elements in solid silicon *J. Alloys Compd.* **490** 31–41

[73] TANG K and Tangstad M 2012 A thermodynamic description of the Si-rich Si-Fe system *Acta Metall. Sin.* **25** 249–55

[74] Liang S-M and Schmid-Fetzer R 2014 Modeling of Thermodynamic Properties and Phase Equilibria of the Si-P System *J. Phase Equilibria Diffus.* **35** 24–35

[75] Lim S S, Rossiter P L and Tibballs J E 1995 Assessment of the Al-Ag binary phase diagram *Calphad* **19** 131–41

[76] Mirković D, Gröbner J, Schmid-Fetzer R, Fabrichnaya O and Lukas H L 2004 Experimental study and thermodynamic re-assessment of the Al–B system *J. Alloys Compd.* **384** 168–74

[77] Kim S S and Sanders T H 2006 Thermodynamic assessment of the metastable liquidi in the Al–In, Al–Bi and Al–Pb systems *Model. Simul. Mater. Sci. Eng.* **14** 1181–8

[78] Ozturk K, Zhong Y, Chen L-Q, Liu Z-K, Sofo J O and Wolverton C 2005 Linking first-principles energetics to CALPHAD: An application to thermodynamic modeling of the Al-Ca binary system *Metall. Mater. Trans. A* **36** 5–13

[79] Liang S-M and Schmid-Fetzer R 2015 Thermodynamic assessment of the Al–Cu–Zn system, part II Al–Cu binary system *Calphad* **51** 252–60

[80] Jacobs M H G and Schmid-Fetzer R 2009 Phase behavior and thermodynamic properties in the system Fe–Al *Calphad* **33** 170–8

[81] Watson A 1992 Re-assessment of phase diagram and thermodynamic properties of the Al-Ga system *Calphad* **16** 207–17

[82] Kaban I, Curiotto S, Chatain D and Hoyer W 2010 Surfaces, interfaces and phase transitions in Al–In monotectic alloys *Acta Mater.* **58** 3406–14

[83] Liang S-M and Schmid-Fetzer R 2013 Thermodynamic assessment of the Al–P system based on original experimental data *Calphad* **42** 76–85

[84] Balakumar T and Medraj M 2005 Thermodynamic modeling of the Mg–Al–Sb system *Calphad* **29** 24–36

[85] Ansara I, Dinsdale A T and Rand M H 1998 *COST 507: Thermochemical Database for Light Metal Alloys* vol 2

[86] Witusiewicz V T, Bondar A A, Hecht U, Rex S and Velikanova T Y 2008 The Al–B–Nb–Ti system III. Thermodynamic re-evaluation of the constituent binary system Al–Ti *J. Alloys Compd.* **465** 64–77

[87] He X C, Wang H, Liu H S and Jin Z P 2006 Thermodynamic description of the Cu–Ag–Zr system *Calphad* **30** 367–74

[88] Wang C P, Guo S H, Tang A T, Pan F S, Liu X J and Ishida K 2009 Thermodynamic assessments of the Cu–B and Cu–Tm systems *J. Alloys Compd.* **482** 67–72

[89] TEPPO O, NIEMELA J and TASKINEN P 1990 AN ASSESSMENT OF THEI THERMODYNAMIC PROPERTIES AND PHASE DIAGRAM OF THE SYSTEM Bi-Cu *Thermochim. Acta* **173** 137–50

[90] Risold D, Hallstedt B and Gauckler L J 1996 Thermodynamic optimization of the Ca-Cu and Sr-Cu systems *Calphad* **20** 151–60

[91] Chen Q and Jin Z 1995 The Fe-Cu system: A thermodynamic evaluation *Metall. Mater. Trans. A* **26** 417–26

[92] Li J-B, Ji L N, Liang J K, Zhang Y, Luo J, Li C R and Rao G H 2008 A thermodynamic assessment of the copper–gallium system *Calphad* **32** 447–53

[93] Liu H S, Liu X J, Cui Y, Wang C P, Ohnuma I, Kainuma R, Jin Z P and Ishida K 2002 Thermodynamic Assessment of the Cu-In Binary System **23** 7

[94] Noda T, Oikawa K, Itoh S, Hino M and Nagasaka T 2009 Thermodynamic evaluation of Cu–Cu3P system based on newly determined Gibbs energy of formation of Cu3P *Calphad* **33** 557–60

[95] Liu X J, Wang C P, Ohnuma I, Kainuma R and Ishida K 2000 Thermodynamic assessment of the phase diagrams of the Cu-Sb and Sb-Zn systems *J. Phase Equilibria* **21** 432–42

[96] Li D, Franke P, Fürtauer S, Cupid D and Flandorfer H 2013 The Cu–Sn phase diagram part II: New thermodynamic assessment *Intermetallics* **34** 148–58

[97] Kumar H, Ansara I, Wollants P and Delaey L 1996 Thermodynamic optimization of the Cu-Ti system *Z. Für Met.* **87** 666–72

[98] Swartzendruber L J 1984 The Ag−Fe (Silver-Iron) system *Bull. Alloy Phase Diagr.* **5** 560–4

[99] Rompaey T V, Hari Kumar K C and Wollants P 2002 Thermodynamic optimization of the B–Fe system *J. Alloys Compd.* **334** 173–81

[100] Boa D, Hassam S, Kotchi K P and Rogez J 2006 Thermodynamic investigation of the moderately dilute liquid Bi–Fe–Sb alloys *Thermochim. Acta* **444** 86–90

[101] Ohno M and Yoh K 2009 Thermodynamic Calculation of Phase Equilibria in As-Fe-In Ternary System Based on CALPHAD Approach *Mater. Trans.* **50** 1202–7

[102] Ohtani H, Hanaya N, Hasebe M, Teraoka S and Abe M 2006 Thermodynamic analysis of the Fe–Ti–P ternary system by incorporating first-principles calculations into the CALPHAD approach *Calphad* **30** 147–58

[103] Huang Y-C, Gierlotka W and Chen S-W 2010 Sn–Bi–Fe thermodynamic modeling and Sn–Bi/Fe interfacial reactions *Intermetallics* **18** 984–91

[104] Bo H, Wang J, Duarte L, Leinenbach C, Liu L, Liu H and Jin Z 2012 Thermodynamic re-assessment of Fe–Ti binary system *Trans. Nonferrous Met. Soc. China* **22** 2204–11

[105] Lee B-Z, Oh C-S and Lee D N 1994 A thermodynamic evaluation of the Ag-Pb-Sb system *J. Alloys Compd.* **215** 293–301

[106] Yu S-K, Sommer F and Predel B 1996 Isopiestic measurements and assessment of the Al-Pb system *Z. Für Met.* **87** 574–80

[107] Idbenali M, Servant C, Selhaoui N and Bouirden L 2008 A thermodynamic reassessment of the Ca–Pb system *Calphad* **32** 64–73

[108] Vaajamo I and Taskinen P 2011 A thermodynamic assessment of the iron–lead binary system *Thermochim. Acta* **524** 56–61

[109] Mathon M, Miane J M, Gaune P, Gambino M and Bros J P 1996 Gallium + lead system: molar heat capacity and miscibility gap *J. Alloys Compd.* **237** 155–64

[110] Ohtani H, Okuda K and Ishida K 1995 Thermodynamic study of phase equilibria in the Pb-Sn-Sb system *J. Phase Equilibria* **16** 416–29

[111] Gierlotka W 2012 Thermodynamic Description of the Quaternary Ag-Cu-In-Sn System *J. Electron. Mater.* **41** 86–108

[112] Ma X, Yoshikawa T and Morita K 2012 Phase relations and thermodynamic property of boron in the silicon-tin melt at 1673K *J. Alloys Compd.* **529** 12–6

[113] Lee B-J, Oh C-S and Shim J-H 1996 Thermodynamic assessments of the Sn-In and Sn-Bi binary systems *J. Electron. Mater.* **25** 983–91

[114] Ohno M, Kozlov A, Arroyave R, Liu Z and Schmidfetzer R 2006 Thermodynamic modeling of the Ca–Sn system based on finite temperature quantities from first-principles and experiment *Acta Mater.* **54** 4939–51

[115] Anderson T J and Ansara I 1992 The Ga-Sn (gallium-tin) system *J. Phase Equilibria* **13** 181–9

[116] Miettinen J 2001 Thermodynamic description of Cu-Sn-P system in the copper-rich corner *Calphad* **25** 67–78

[117] Manasijević D, Vřešt`ál J, Minić D, Kroupa A, Živković D and Živković Ž 2008 Experimental investigation and thermodynamic description of the In–Sb–Sn ternary system *J. Alloys Compd.* **450** 193–9

[118] Yin F, Tedenac J-C and Gascoin F 2007 Thermodynamic modelling of the Ti–Sn system and calculation of the Co–Ti–Sn system *Calphad* **31** 370–9

[119] Gómez-Acebo T 1998 Thermodynamic assessment of the Ag-Zn system *Calphad* **22** 203–20

[120] Chen S-L and Chang Y A 1993 A thermodynamic analysis of the Al-Zn system and phase diagram calculation *Calphad* **17** 113–24

[121] Chen Z, Yin F, Zhao M and Li Z 2013 Experimental Investigation and Thermodynamic Calculation of the B-Co-Zn Ternary System *J. Phase Equilibria Diffus.* **34** 366–74

[122] Djaballah Y, Bennour L, Boubarkat F and Belgacem-Bouzida A 2005 Thermodynamic assessment of the binary system (Bi–Zn) *Model. Simul. Mater. Sci. Eng.* **13** 361–9

[123] Brubaker C O and Liu Z-K 2001 A computational thermodynamic assessment of the Ca-Zn system *Calphad* **25** 381–90

[124] Wang J, Xu H, Shang S, Zhang L, Du Y, Zhang W, Liu S, Wang P and Liu Z-K 2011 Experimental investigation and thermodynamic modeling of the Cu–Si–Zn system with the refined description for the Cu–Zn system *Calphad* **35** 191–203

[125] Nakano J, Malakhov D V and Purdy G R 2005 A crystallographically consistent optimization of the Zn–Fe system *Calphad* **29** 276–88

[126] Mathon M, Jardet K, Aragon E, Satre P and Sebaoun A 2000 Al-Ga-Zn System: Reassessments of the Three Binary Systems and Discussion on Possible Estimations and on Optimisation of the Ternary System *Calphad* **24** 253–84

[127] Lee B TIG;RMODYNAMIC ASSESSMENTS OF THE Sn-Zn AND In-Zn BINARY SYSTEMS 10

[128] Liu Y L, Zhou B, Lv W, Wu C, Su X and Wang J 2016 Experimental investigation and thermodynamic assessment of the Zn–Si–P system *Surgace Coat. Technol.* **206** 370–7

[129] Li J-B, Record M-C and Tedenac J-C 2007 A thermodynamic assessment of the Sb–Zn system *J. Alloys Compd.* **438** 171–7

[130] Doi K, Ono S, Ohtani H and Hasebe M 2006 Thermodynamic study of the phase equilibria in the Sn−Ti−Zn ternary system *J. Phase Equilibria Diffus.* **27** 63–74

[131] Weiss T and Schwerdtfeger K 1994 Chemical equilibria between silicon and slag melts *Metall. Mater. Trans. B* **25** 497–504

[132] Teixeira L A V, Tokuda Y, Yoko T and Morita K 2009 Behavior and State of Boron in CaO–SiO2 Slags during Refining of Solar Grade Silicon *ISIJ Int.* **49** 777–82

[133] Teixeira L A V and Morita K 2009 Removal of Boron from Molten Silicon Using CaO–SiO2 Based Slags *ISIJ Int.* **49** 783–7

[134] Jakobsson L K and Tangstad M 2014 Distribution of Boron Between Silicon and CaO-MgO-Al2O3-SiO2 Slags *Metall. Mater. Trans. B* **45** 1644–55

[135] Li M, Utigard T and Barati M 2014 Removal of Boron and Phosphorus from Silicon Using CaO-SiO2-Na2O-Al2O3 Flux *Metall. Mater. Trans. B* **45** 221–8

[136] Jakobsson L K and Tangstad M 2015 Thermodynamic Activities and Distributions of Calcium and Magnesium Between Silicon and CaO-MgO-SiO2 Slags at 1873 K (1600 °C) *Metall. Mater. Trans. B* **46** 595–605

[137] Ahn S H, Jakobsson L K and Tranell G 2017 Distribution of Calcium and Aluminum Between Molten Silicon and Silica-Rich CaO-Al2O3-SiO2 Slags at 1823 K (1550 °C) *Metall. Mater. Trans. B* **48** 308–16

[138] Kume K, Morita K, Miki T and Sano N 2000 Activity Measurement of CaO-SiO2-AlO1.5-MgO Slags Equilibrated with Molten Silicon Alloys *ISIJ Int.* **40** 561–6

[139] Noguchi R, Suzuki K, Tsukihashi F and Sano N 1994 Thermodynamics of boron in a silicon melt *Metall. Mater. Trans. B* **25** 903–7

[140] Sunkar A S and Morita K 2009 Thermodynamic Properties of the MgO–BO1.5, CaO–BO1.5, SiO2–BO1.5, MgO–BO1.5–SiO2 and CaO–BO1.5–SiO2 Slag Systems at 1 873 K *ISIJ Int.* **49** 1649–55
